# Endocardial transcription factor HAND2 orchestrates hypoxic and TGF-**β** signaling to modulate coronary artery formation

**DOI:** 10.1172/JCI203310

**Published:** 2026-08-06

**Authors:** Huijuan Wang, Haosheng Zhang, Leiyin Zheng, Peihan Zhang, Yuqian Wang, Sijia Ding, Wenping Liu, Yuanming Cheng, Zhongzhou Yang, Wen Luo

**Affiliations:** 1 Institute of Modern Biology (IMB), Department of Vascular Surgery, Cardiovascular Medical Center, and; 2 IMB, Department of Hematology, Nanjing Drum Tower Hospital, The Affiliated Hospital of Nanjing University Medical School, Nanjing University, Nanjing, China.; 3 State Key Laboratory of Pharmaceutical Biotechnology, Nanjing University, Nanjing, China.; 4 Jiangsu Key Laboratory of Molecular Medicine, Nanjing University Medical School, Nanjing University, Nanjing, China.

**Keywords:** Cardiology, Vascular biology, Cardiovascular disease

## Abstract

The endocardium is a major source of coronary angiogenesis and arterialization, through coordinated cell fate transition and migration. However, the transcriptional regulatory network synchronizing cell fate determination and movement remains unclear. Here, we identified transcription factor HAND2 as a key candidate for coronary vascular formation. Endocardial deletion of *Hand2* in mice disrupted arterial-venous networks and stunted coronary arteries, paralleling a ventricular noncompaction phenotype. Moreover, deletion of *Hand2* produced excessive tip cells with defective movement. RNA-seq analysis revealed enhanced hypoxic metabolic activation but declined TGF-β/p38MAPK–dependent endothelial-mesenchymal transition. In contrast, genetic inhibition of the core hypoxic regulators or pharmaceutical administration of TGF-β2 partially recovered the coronary arterial defects in *Hand2* mutants. Furthermore, HAND2 was found to directly bind to promoters of the target genes, harmonizing cell migration and cell fate transition. These findings pinpoint HAND2 as an essential regulator of the endocardial transcriptional regulatory network for coronary arterialization and provide potential therapeutic targets for coronary artery diseases.

## Abstract 2

Key cardiac transcription factor HAND2 helps build coronary arteries, revealing potential therapeutic targets for congenital and ischemic heart disease.

## Graphical abstract

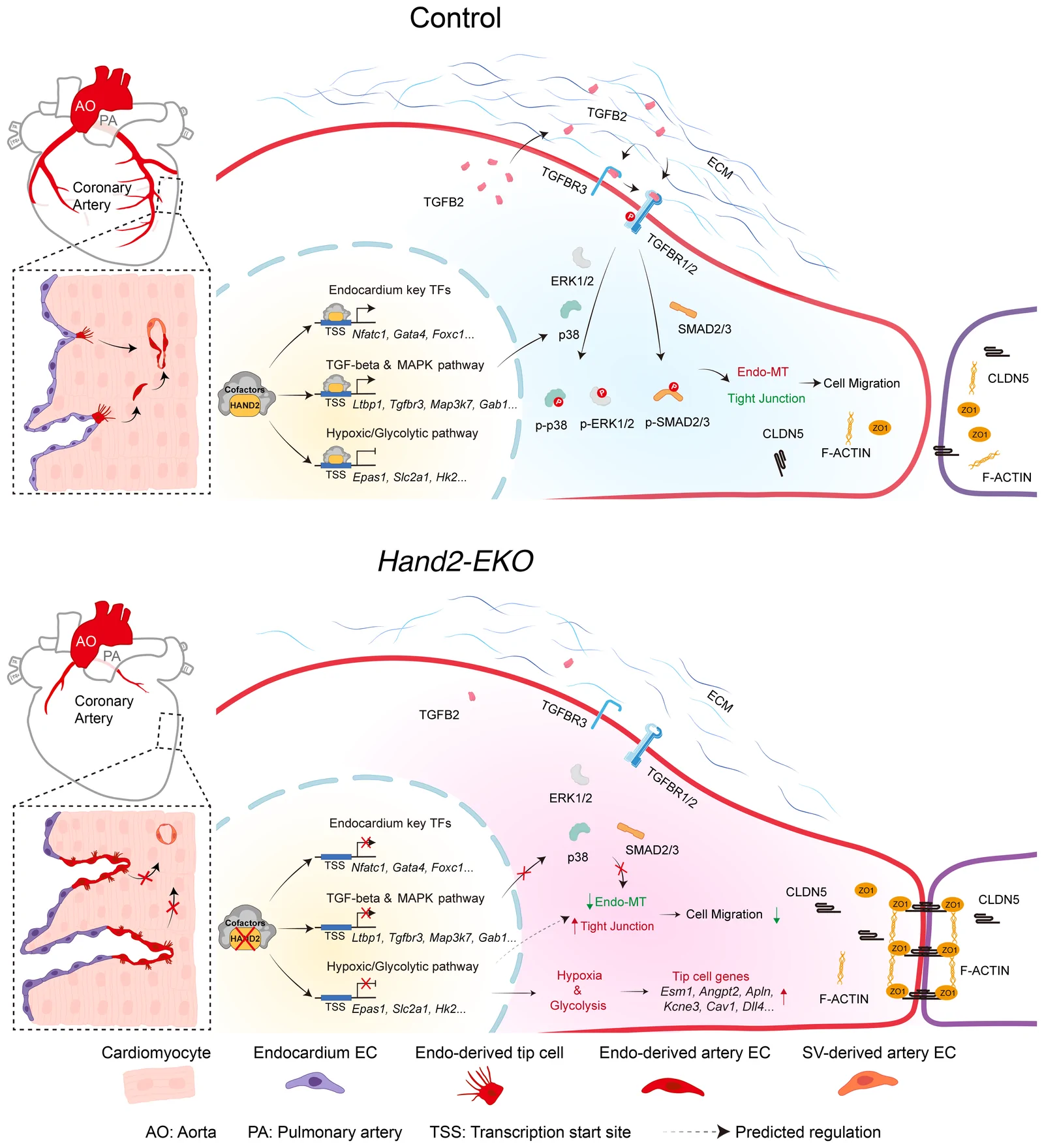

## Introduction

During embryonic development, a large number of endocardial cells occupy the vast inner surface of the heart to form the endocardium (Endo), making contributions to multiple cell lineages in the developing heart, such as mural cells, macrophages, fibroblasts, myeloid cells, adipocytes, and coronary endothelial cells (ECs) (1–5). Through endothelial-mesenchymal transition (Endo-MT), the endocardial cells change cell fate to mesenchymal cells that subsequently remodel into cardiac valves and septa and also give rise to vessel-associated pericytes, smooth muscle cells, and coronary ECs. Thus, endocardial cells demonstrate great plasticity, which is a key prerequisite for cell fate specification and cardiac morphogenesis (6, 7). Disruption of endocardial cell fate transition results in severe consequences, leading to a series of congenital heart diseases and postnatal cardiomyopathy (6, 8).

An interplay exists between the endocardial and myocardial cells (cardiomyocytes) during heart development (9–11). In response to the signaling from the myocardium and tissue microenvironment, endocardial cells undergo Endo-MT to generate mesenchymal cells that afterward acquire a tip cell–like feature. These cells migrate toward the intramyocardial layer to form endocardial tunnels and eventually adopt arterialization for development of coronary arteries (12, 13). Both the chamber/ventricular coronary arteries and the interventricular septal arteries are derived from the endocardial cells (14, 15). It is known that several signaling pathways and angiocrines are involved in regulating these processes, including NOTCH, VEGF, BMP, TGF, and CXCL12 (11, 13, 16–22). Nonetheless, it is still elusive how these signals are monitored and coordinated by the gene regulatory network (GRN) mediated by Endo-enriched transcription factors (TFs).

Although HAND2 acts as a key regulator of cardiac structures and morphology (23), the role of *Hand2* in coronary vessel development remains unclear. Previous studies showed that *Hand2* Endo-specific deletion with *Nfatc1-Cre* caused embryonic lethality at approximately E14.0 (24), hindering the exploration of the role of *Hand2* in coronary vessel development at the late stage. Furthermore, a recent scRNA-seq study of E11.5 endocardial ECs revealed that deletion of *Hand2* disrupts numerous endocardial GRNs and identified the novel HAND2 target gene, *Klf2*, a shear-stress master regulator (25). Still, it remains unclear how HAND2 is coupled with other regulators to control arterial-venous differentiation. There is therefore great interest in elucidating the role of *Hand2* in coronary angiogenesis and arterialization.

Here, we analyzed the TFs from sinus venosus–derived (SV-derived) and Endo-derived ECs and identified HAND2 as an Endo-enriched regulator that orchestrates endocardial GRNs and is critical for coronary artery development and ventricular compaction. We uncovered a dual role for HAND2 in coupling hypoxic metabolic regulation with TGF-β/p38MAPK–dependent Endo-MT and extracellular matrix (ECM) remodeling in endocardial cells for adoption of arterial cell fate and coronary artery formation. At the molecular level, HAND2 binds to the promoters of the key genes involved in the above biological processes to coordinate their expression and subsequently controls normal coronary artery formation.

## Results

### Gene signature analysis of distinct cardiac endothelial populations and identification of the core endocardial TFs.

The two distinct cellular origins of coronary endothelium, the Endo and the SV, make distinct contributions to cardiac coronary formation. While SV derivatives give rise to the dorsal myocardium, the endocardial derivatives contribute to the ventral portions of the heart and ventricular septum (12, 26). Nonetheless, the key regulators or transcriptional regulatory networks to control the fate conversion of SV and endocardial cells remain unclear.

To identify the critical regulators for coronary vascular development, we performed analysis of the RNA-seq data of isolated coronary ECs and endocardial cells from mouse heart at E14.5 (the key stage for arterial differentiation) (27). Gene Ontology (GO) term enrichment and Kyoto Encyclopedia of Genes and Genomes (KEGG) pathway analysis displayed upregulation and enrichment of the gene signatures involved in metabolism (e.g., glycolysis, mTOR, lysosome, AMPK), angiogenesis (ECM, TGF-β, ErbB, VEGF), and cell cycle as well as Wnt signaling pathway, in endocardial population, whereas those implicated in inflammatory (e.g., NF-κB, TNF, JAK-STAT, cytokine, PPAR, cAMP), Apelin, FoxO, MAPK, HIF, and NOTCH signaling pathways were enriched in coronary EC population. These findings suggested that SV- and Endo-derived cells incorporate and establish coronary vasculature in response to diverse signaling stimulations, while also modulating cardiac septation, structural morphogenesis, and contractility (Figure 1, A and B). Owing to the high correlation between the GRN and signaling activation, we spatially analyzed a series of key cardiac TFs in the SV and Endo based on the RNA-seq data. Interestingly, these core TFs in the endothelium were more enriched in the Endo than in the coronary endothelium (Figure 1C), suggesting the high plasticity and multidifferentiation potential of endocardial cells during cardiovascular development. We further identified *Hand2* and *Gata4* as the top TFs exclusively expressed in endocardial cells of the endothelium (Figure 1D). Immunostaining revealed the specific colocalization of HAND2 with ERG (an endothelial marker) in the Endo of the developing heart from E12.5 to E17.5 (Figure 1E). These findings suggest the unique role of HAND2 and GATA4 in regulating endocardial cell fate conversion and coronary artery development.

### Endothelial Hand2 deletion impairs coronary vessel development.

Previous study has shown that endocardial deletion of *Hand2* using *Nfatc1-Cre* results in embryonic lethality by E14.0. These mice displayed tricuspid atresia, multiple ventricular septa, and hypervascularized myocardium (24), which prevented investigating the details of coronary vessel development and remodeling at late developmental stage. To overcome this obstacle and study the role of *Hand2* in coronary development, we intercrossed *Hand2*-floxed mice with the inducible EC-specific *Cdh5-CreERT2* mice to generate *Hand2* EC-specific knockout alleles (*Hand2-EKO*). Tamoxifen was administered at E11.5–12.5 when the coronary vascular plexus began to emerge on the surface of the heart, and embryos were collected at E15.5 (Figure 2A). We first verified Cre recombination and *Hand2* deletion efficiency using an antibody against HAND2 (Figure 2B) and observed the absence of HAND2^+^ERG^+^ double-positive cells in the Endo of *Hand2-EKO* mice (Figure 2C), a finding that was further verified in *Hand2-EKO; iSuRe-Cre* mice (*iSuRe-Cre*, inducible dual reporter-Cre) (28) (Supplemental Figure 1, A and B; supplemental material available online with this article; https://doi.org/10.1172/JCI203310DS1). The *Hand2* deletion mice were embryonic lethal at approximately E16.0, exhibiting edema and hemorrhage (Figure 2D). Histological analysis with H&E staining, and immunofluorescence (IF) staining for EMCN, showed marked thinning of the ventricular myocardial wall with excessive trabeculae in the *Hand2-EKO* hearts. This hypertrabeculation/noncompaction phenotype indicates that endocardial *Hand2* is required for myocardial development and meanwhile suggests of participation in the crosstalk between ECs and cardiomyocytes (Figure 2, E–G).

To test the function of endothelial HAND2 in coronary vessel development, we performed whole-mount IF staining of the coronary veins and arteries in control and *Hand2-EKO* mice. In control embryos, the coronary vasculature spread on the dorsal side of the hearts to establish a mature venous network (Figure 2H). However, in *Hand2-EKO* embryos, coronary venous plexus sprouting and branching were stunted, and vessel density and diameter of large veins were profoundly reduced, together with a significant decrease in the percentage of ERG^+^ cells in the vessels (Figure 2, H and I). Moreover, IF staining of prearterial markers, DLL4 and UNC5B, demonstrated substantially enhanced numbers of arterial capillary, in parallel with reduction of CX40, JAG1, and SM22, the matured arterial markers. Consistently, the large coronary arteries demonstrated a massive decrease in lumen size and defective remodeling and expansion in *Hand2-EKO* mice (Figure 2, J and K, and Supplemental Figure 1, C–E).

Taken together, these results indicate a crucial role of the endothelial HAND2 in coronary vessel formation and arterialization.

### Endothelial Hand2 deletion disrupts cell proliferation and ventricle compaction.

Abnormal coronary angiogenesis causes defective ventricular chamber development (22, 29–31). The above results suggest that endothelial *Hand2* deletion leads to the ventricular noncompaction phenotype, we therefore examined myocardial trabeculation and compaction. We first employed a 5-ethynyl-2′-deoxyuridine (EdU) incorporation assay to measure cardiomyocyte proliferation in compact and trabecular layers (Supplemental Figure 1F). Quantification of NKX2.5^+^ and EdU^+^ double-positive cells revealed that *Hand2-EKO* mice had decreased cardiomyocyte proliferation in the compact layer but inversely enhanced proliferation in the trabecular layer (Supplemental Figure 1, G and H). Moreover, IF staining for α-actinin, a microfilament protein that mediates cardiac sarcomeric protein interaction, exhibited highly organized sarcomeric structure in the trabecular layer compared with the compact layer (myocardial wall) in controls (Supplemental Figure 1I). However, this type of well-organized sarcomeric structure was observed in intermediate myocardium of *Hand2-EKO* hearts (Supplemental Figure 1I). These noncompaction features were further supported by qPCR assay and IF staining that showed downregulation of compact myocardial marker genes (*Mycn*, *Hey2* and *Tbx20*) but enhancement of trabecular myocardial marker genes (*Bmp10*, *Gja5* and *Nppb*) (Supplemental Figure 1, J and K). Furthermore, we detected increased cell proliferation of ECs in the trabecular layer but decreased cell proliferation of ECs in the compact layer (Supplemental Figure 1, L and M).

Both the Endo and the coronary vasculature affect myocardial compaction (22, 29). To specifically understand endocardial HAND2 regulation on myocardial development, we delayed induction of *Hand2* deletion at E13.5–14.5, when ECs passed the periods of sprouting and early specification, and harvested embryos at E17.5 (Supplemental Figure 2A). Histological analysis revealed thinning of the compact layer and thickening of the trabecular layer in *Hand2* mutants, which was further visualized by IF staining for EMCN (Supplemental Figure 2, B and C). In addition, we detected increased cell proliferation of cardiomyocytes and ECs in the trabecular layer (Supplemental Figure 2, D–G). IF staining for BMP10 and CX40, two well-known trabecular markers, revealed abnormally extended expression into compacted myocardium (Supplemental Figure 2H). Strikingly, endothelial *Hand2* deletion reduced the expression of several noncompaction-associated genes, including *Ptbp1*, *Casz1*, *Jarid2*, *Prdm16*, *Myh7*, and *Nrg1*, whose deletion/mutation would cause ventricular noncompaction phenotype (21, 32, 33) (Supplemental Figure 2I). Although *Hand2-EKO* hearts showed changes in ventricular patterning, coronary artery remodeling, and maturation were not affected (Supplemental Figure 2J).

Collectively, these results indicate that cardiac endothelial *Hand2* influences ventricular chamber development through both endocardial and coronary vascular regulation on myocardial development.

### Endocardial Hand2 deficiency impaired coronary arterial formation.

To more precisely explore the function of endocardial HAND2 in coronary vascular and cardiac chamber development, we crossed *Bmx-CreERT2* mice, in which Cre is specifically expressed in the Endo and arterial cells, with *Hand2*-floxed mice to generate *Bmx-CreERT2; Hand2 ^fl/fl^* (*Hand2-EKO^Bmx^*) mice. Deletion of *Hand2* was induced at E10.5–11.5 by administration of 4-hydroxy-tamoxifen (4-OH), and the embryos were harvested at E15.5 (Figure 3A). Cre expression and *Hand2* deletion efficiency were examined by IF imaging of TdTomato^+^ and staining for ERG^+^HAND2^+^ in the Endo (Figure 3, B and C) and were also tested using isolated endocardial ECs for *Hand2* expression through qPCR assay (Figure 3D). Histological analysis of the hearts displayed poorly developed hearts, including thinned myocardial wall but thickened trabecular layer, a phenotype of ventricular noncompaction in *Hand2-EKO^Bmx^* mice (Figure 3, E–H). Furthermore, we detected less coronary artery extension and remodeling with normal coronary veins in *Hand2-EKO^Bmx^* mice (Figure 3, I–L).

Next, we generated *Hand2-EKO^Pdgfb^* mice, which selectively inactivated *Hand2* in SV-derived coronary ECs. Deletion of *Hand2* was induced at E11.5–12.5 by administration of tamoxifen, and the embryos were collected at E15.5 or E17.5 (Supplemental Figure 3A). As expected, no obvious phenotypes were observed in the *Hand2-EKO^Pdgfb^* mice (Supplemental Figure 3, B–G).

Overall, these results show that endocardial HAND2 plays a predominant role in cardiac arterialization and chamber development.

### Spatiotemporal gene expression changes in Hand2-EKO mice impairs endocardial angiogenesis.

To understand the regulatory mechanisms of HAND2 in the Endo, we performed bulk and single-cell transcriptomics analysis of the ECs in *Hand2-EKO* mice. Since the *Cdh5-creERT2* line shows more robust *Hand2* recombination efficiency than the *Bmx-creERT2* line (Figure 3D and Supplemental Figure 7A), we assumed that *Hand2-EKO* (namely *Hand2-EKO^Cdh5^*) is an ideal model for studying the role of *Hand2* in Endo. We isolated CD31^+^TdTomato^+^ cardiac ECs from E14.5 *Cdh5-CreERT2; iSuRe-Cre* controls and *Hand2-EKO; iSuRe-Cre* mutants after tamoxifen induction at E11.5–12.5 (Figure 4A). We detected significant downregulation of both arterial marker genes (*Slc45a4*, *Gja4*, *Gja5*, *Cxcl12*, *Hey1*) and venous marker genes (*Tbx20*, *Ptgs1*, *Wnt2*, *Aplnr*, *Ephb4*), as well as Endo-enriched marker genes (*Adgrg6*, *Blvrb*, *Irx5*, *Tmem108*, *Npr3*, *Nrk, Igf2r, Ece1, Plvap*) in *Hand2-EKO* mice (Figure 4, B and C). In contrast, the SV-enriched marker genes (*Pdgfb*, *Fabp4*, *Kitl*, *Cd36*, *Jag2*, *Pcdh17, Dhah1*) were profoundly enhanced in these mice (Figure 4C). IF staining further demonstrated that reduced expression of endocardial-specific marker PLVAP and ectopic expression of SV-enriched protein FABP4 in the Endo of *Hand2* mutant mice (Figure 4D). RNA-seq data revealed a distinct gene expression profile, with 1,586 genes downregulated and 1,496 genes upregulated (*q* < 0.05, |log_2_FC|> 0.25) (Supplemental Figure 4A). GO terms and KEGG pathway analysis of differentially expressed genes showed that several gene signatures involved in hypoxia and metabolism (p53 and HIF-1 pathway and glycolysis) were upregulated, whereas those signatures implicated in angiogenesis (Endo-MT, MAPK, and TGF-β pathways) were downregulated (Supplemental Figure 4, B and C).

In addition, we performed single-cell RNA-seq (scRNA-seq) analysis on isolated CD31^+^TdTomato^+^ ECs from control and *Hand2-EKO* mice. These double-positive ECs were subsequently classified to be endocardial cells, capillaries derived from the Endo (Endo-Cap), prearterial cells, arterial cells, and prevenous cells, with well-accepted specific markers (Figure 4, E and F). Module scores were calculated and compared with the reported scRNA-seq datasets that served as quality controls (Supplemental Figure 5, A–C). Moreover, a systematic comparative bioinformatic evaluation validated the lineage trajectory rather than the cell-state transitions that were altered in *Hand2-EKO* mice (Supplemental Figure 5, D–H). UMAP analysis confirmed decreased *Hand2* expression in Endo (Figure 4G). Cell cluster analysis uncovered augmented cell numbers of Endo-Cap clusters in *Hand2-EKO* mice compared with controls, suggesting of high potency to differentiate into coronary arteries (Figure 4E). A compensatory increase in cell numbers of prearterial cell and prevenous cell clusters from SV-derived ECs in *Hand2-EKO* mice was also identified (Figure 4E). Interestingly, the Endo-Cap cluster exhibited high expression of tip cell–enriched markers but reduced Endo-MT-associated gene expression (Figure 4, H and I). GO enrichment analysis further revealed that TGF-β and hypoxia pathways were involved in HAND2-mediated cell fate conversion (Figure 4J). Furthermore, scRNA-seq analysis also revealed ectopic expression of SV-enriched genes in *Hand2*-deficient Endo and reduction of Endo- and arterial-enriched genes (Supplemental Figure 4, G–J), which is consistent with the bulk RNA-seq results. These findings indicate a reciprocal compensation between SV and Endo-derived vessels during coronary development.

In *Hand2-EKO* hearts, we found that expression of cell adhesion proteins E-cadherin and ZO-1 was enhanced, while that of vimentin, a well-known mesenchymal marker, was downregulated (Figure 4, K–M). Western blotting and qPCR analysis disclosed disrupted Endo-MT in *Hand2-EKO* hearts and isolated ECs (Figure 4N and Supplemental Figure 4D). For instance, SNAIL-1, a downstream target of HAND2 involved in cardiac cushion mesenchyme formation (34), and SLUG, another well-known Endo-MT marker (35), were reduced in the Endo of *Hand2-EKO* mice (Figure 4N), suggesting a conserved mechanism for Endo-MT regulation. Moreover, we detected that several genes encoding ECM proteins (36), including *Col1a1*, *Col3a1, Col6a1, Postn*, *Olfml2a, Tgfbi*, and *Ecm2*, were downregulated in *Hand2-EKO* cells (Supplemental Figure 4E).

To further determine whether endocardial *Hand2* regulates coronary angiogenesis through Endo-MT, we performed a ventricular explant assay. The ventricles from control (*Bmx-creERT2* line only) and *Hand2-EKO^Bmx^* mice coupled with the *iSuRe-Cre* reporter allele at E12.0 were dissected for in vitro ventricular explant culture (Figure 4O). Compared with controls, *Hand2-EKO^Bmx^* explants showed a significant decrease in vessel sprouting and branching (Figure 4P). We next performed an in vivo lineage-tracing experiment to assess *Hand2*-mediated migration of endocardial ECs into myocardium. 1.5 mg 4-OH tamoxifen was given to *Bmx-CreERT2; iSuRe-Cre* reporter mice and *Hand2-EKO^Bmx^; iSuRe-Cre* reporter mice for monitoring cell migration. Normally, endocardial ECs could ingress and integrate into the coronary artery of the myocardium, but this migration failed in *Hand2* mutants, indicating that HAND2 positively controls EC migration in the Endo (Figure 4Q).

Taken together, these results demonstrate that endocardial HAND2 is required for Endo-MT and regulation of coronary angiogenesis in the developing heart.

### Induction of hypoxia and tip cell signatures in Hand2-EKO mice.

Gene set enrichment analysis of our RNA-seq data and the data from E9.0–E9.25 *Hand2-*knockout atrioventricular canals revealed an overlapping coverage of hypoxia- and glycolysis-associated genes in *Hand2* mutants (34) (Supplemental Figure 6, A and B). Subsequently, we examined the expression of key TFs HIF1α and HIF2α in *Hand2-EKO* mice. IF staining showed that the expression of both HIF1α and HIF2α, as well as their downstream targets, was increased upon loss of *Hand2* in Endo (Figure 5, A and B), suggesting that HAND2 acts upstream to suppress HIF pathway. Accordingly, glycolysis genes were upregulated at both mRNA and protein levels in *Hand2-EKO* mice (Figure 5, C and D). Upon exposure to hypoxia, the expression of p53 and its downstream target genes in regulating cell cycle were enhanced in *Hand2-EKO* mice (Supplemental Figure 4F). Strikingly, functional augmentation of the hypoxia pathway through endocardial deletion of *Vhl*, a negative modulator of HIF pathway, recapitulates cardiac and coronary vascular defects observed in *Hand2-EKO* mice (Supplemental Figure 6, C–E).

Previous studies have shown that endocardial hypoxia promotes coronary angiogenesis, followed by upregulation of hypoxia-related genes in ESM1-expressing intramyocardial tip cells during heart development (13, 37). To ascertain whether HAND2 is involved in this process, we checked the expression of tip cell markers and found a significant upregulation of tip cell–enriched genes, including *Esm1*, *Angpt2*, *Apln*, *Kcne3*, *Mcan*, *Cav1*, *Dll4* and *Flt4,* in total cardiac ECs and isolated EMCN^+^ endocardial cells in *Hand2-EKO* hearts (Figure 5, E and F, and Supplemental Figure 6F). Interestingly, IF staining for tip cell–enriched proteins such as ANGPT2, vWF, and CAV1, revealed prominent accumulation in tip cell–like endocardial cells showing restricted migration in the Endo in *Hand2-EKO* mice (Figure 5G). Meanwhile, we observed decreased coverage of mural cells such as pericytes (PDGFRB^+^, NG2^+^, and OLFML3^+^ cells) and smooth muscle cells (aSMA^+^ cells) in *Hand2-EKO* mice (Supplemental Figure 6G). These results suggest that augmentation of HIF pathway could be responsible for compromised coronary development in *Hand2-EKO* mice. Thus, we examined the coronary arterialization and cardiac morphological changes in control, *Hand2-EKO*, *Hand2-EKO; HIf1α^Het^,* *Hand2-EKO; HIf2α^Het^*, and *Hand2-EKO; HIf1α/2α^Het^* mice. Notably, suppression of HIF pathway was sufficient to reverse the defects in artery formation and myocardial development caused by endocardial *Hand2* deletion (Figure 5H, and Supplemental Figure 6H). Furthermore, qPCR and IF staining revealed that the expression of tip cell markers and hypoxic/glycolytic pathway was significantly attenuated in ECs from *Hand2/HIf1α/2α* triple mutants, in parallel with increased Endo-MT and ECM remodeling (Supplemental Figure 7, A–G). We also confirmed this genetic (and phenotypic) rescue by abolishing the HIF pathway in *Hand2-EKO^Nfatc1^* mice. We found that cardiac structures were recovered and embryonic survival was enhanced in *Hand2-EKO^Nfatc1^; HIf1α^Het^* and *Hand2-EKO^Nfatc1^; HIf2α^Het^* embryos compared with *Hand2-EKO^Nfatc1^* embryos (Supplemental Figure 6, I–K).

Altogether, these findings strongly suggest that endocardial *Hand2* regulates coronary vascular EC dynamics, migration, and artery formation, acting upstream of hypoxic and glycolytic pathways.

### HAND2 harnesses TGF-β and MAPK-p38 signaling pathway to modulate EC migration and arterialization.

We next performed the CUT&Tag assay to identify the putative binding targets of the HAND2 protein. For this purpose, CD31^+^Tdtomato^+^ ECs were collected from E14.5 controls and *Hand2-EKO* embryonic hearts (Figure 6A). A heatmap indicated a sharp reduction of the signals in *Hand2* deletion mice compared with controls (Figure 6B). Analysis of the genomic location distribution revealed profound distribution of HAND2 in the promoter regions, which accounts for approximately 74% of the peaks (Figure 6C).

Integration of RNA-seq and CUT&Tag datasets identified 304 directly upregulated genes and 351 directly downregulated genes in *Hand2-EKO* mice (Figure 6D). KEGG combined with GO term analysis revealed that gene signatures of p53, HIF-1 pathway, and glycolysis were upregulated, whereas those of MAPK and TGF/SMAD pathway were downregulated. These downregulated genes were highly relative to heart morphogenesis and mesenchymal cell differentiation and are crucial for coronary vessel development (Figure 6E). CUT&Tag and qPCR analysis confirmed these changes (Figure 6F). Next, we examined the phosphorylation level of SMAD2/3, ERK, p38, and JNK, the downstream effectors of TGF-β pathway by Western blotting analysis, and the results demonstrated decreased levels in *Hand2-EKO* hearts. IF staining for TGF-β pathway components (TGF-β2 and TGF-BR3, TAB1 and p-SMAD2/3) showed reduced levels in the Endo of *Hand2-EKO* mice (Figure 6, G and H). Notably, treatment of *Hand2-EKO* embryos with recombinant TGF-β2 protein could restore the defects of myocardium and coronary vessels in a dose-dependent manner (Figure 6, I and J). ECs from *Hand2* mutants treated with TGF-β2 regained expression of arterial-venous and EMT/ECM-associated gene programs, concomitant with a reduction in tip cell–enriched gene expression (Supplemental Figure 7, J–O). Additionally, tip cells from the Endo could migrate into the myocardium as a result of increased Endo-MT (Supplemental Figure 7, P–Q). Since TGF-β–induced activation of p38MAP kinase is critical for EMT (38), we also found recovery of TGF-β and p38MAPK activity when suppressing the HIF pathway, indicating that HIF was also involved in TGF-β and p38MAPK-associated Endo-MT, mediated by HAND2 (Supplemental Figure 7, H and I).

We also uncovered, in the isolated *Hand2*-deficient endocardial ECs, substantially reduced expression levels of multiple key cardiac TFs, including *Gata4*, *Gata5*, *Gata6*, *Nfatc1*, *Foxc1*, and *Foxc2*, which were confirmed by qPCR and IF staining analysis (Supplemental Figure 8, A–C). Strikingly, our CUT&Tag analysis revealed a direct binding of HAND2 on the promoters of these genes to downregulate their transcription (Supplemental Figure 8D). Interestingly, CUT&Tag analysis also showed that HAND2 modulates HIFs and glycolytic-associated gene transcription (Supplemental Figure 8E), which is consistent with ChIP-seq data in *Hand2*-deficient atrioventricular canals (34) (Supplemental Figure 6B), and *Hand2* deficiency resulted in upregulation of these genes (Figure 5B). To confirm the upstream feature of *Hand2*, we found that endothelial/endocardial *Gata4* deletion exhibited similar defective coronary development to that obtained in *Hand2-EKO* hearts (Supplemental Figure 9, A–K).

To further investigate whether HAND2 participates in EC migration and arterial specification in vitro, we induced *HAND2* expression in HUVECs (*HAND2-OE*) (Supplemental Figure 10, A–C). Ectopic expression of *HAND2* increased cell migration and showed a high tube-forming ability (Supplemental Figure 10, D and E). Notably, *HAND2-OE* HUVECs showed upregulated tip cell (*ESM1*, *ADM*, *ANGPT2*, *PDGFB*, and *FLT4*) and arterial (*CD44*, *CXCR4*, *JAG1*, *SOX17*, *EPHB2*, *VEGFA*, *NRP1*, and *SEMA3G*) marker genes, indicating that high *HAND2* expression promotes cell migration and arterial EC fate in vitro and could uncover an insight into artery formation during injury (Supplemental Figure 10F).

Collectively, these results demonstrate that HAND2 acts as an upstream regulator via transcriptional activation of signaling pathways, pinpointing the crucial role of HAND2 to establish the endocardial GRN for cardiac morphogenesis and coronary arterialization.

## Discussion

In the developing heart, coronary arterial ECs differentiate and migrate from Endo in response to hypoxia and other factors secreted from cardiomyocytes. However, the underlying transcriptional regulatory mechanisms of Endo-derived arterialization remain to be elucidated. We found large differences in gene expression profiling between E14.5 SV-derived and Endo-derived ECs, which exhibited distinct gene signatures and functions that precisely regulate coronary angiogenesis and cardiac morphology separately.

We identified the cardiac TF HAND2 and its partner GATA4 (34, 39, 40)*,* specifically expressed in the Endo; they are pivotal regulators of gene transcriptional regulatory network for coronary artery formation and myocardial development. We demonstrate that EC-specific deletion of *Hand2* causes severe defects, including abnormal coronary artery formation and a ventricular noncompaction phenotype, leading to embryonic lethality. Transcriptomic analysis revealed that the spatiotemporal gene expression profiles were disrupted in Endo-derived and SV-derived ECs of *Hand2*-deleted hearts, which were also accompanied by decreases in arterial and venous gene expression. We found that *Hand2* loss upregulates hypoxic and metabolic genes while downregulating TGF-β/p38MAPK–dependent Endo-MT and ECM pathways. Mechanistically, HAND2 directly binds to gene promoters to suppress the hypoxic pathway while activating TGF-β/p38MAPK–dependent Endo-MT and ECM remodeling. This dual regulation facilitates the migration of Endo-derived ECs and their differentiation into arterial cells. Consequently, *Hand2* deletion disrupts this balance, upregulating metabolic genes but inhibiting essential migration signals, leading to defective coronary arteries and a noncompaction cardiomyopathy-like phenotype (Figure 7). Finally, we showed that modulating the hypoxia pathway or administering TGF-β2 can partially rescue these defects. Our study establishes endocardial *Hand2* as an essential upstream regulator of coronary development and suggests its therapeutic potential for promoting neovasculogenesis.

The role of *Hand2* in early heart development has been well-characterized in numerous studies (23). Both *Hand2* systemic knockout and *Hand2* cell type–specific deletion resulted in severe cardiac morphological defects, including hypoplasia of the RV, dilated aortic sac, ventricular septal defects, disorganized endocardial cushions, valvular defects, and reduced trabeculation due to abnormal cell proliferation, differentiation, and migration, indicating that HAND2 plays a vital role in regulating cardiomyocytes, cardiac neural crest cells, ECs, and epicardial precursor cells during cardiac morphogenesis (24, 25, 34, 41–47). In turn, enforced HAND2 expression in cardiomyocytes resulted in the absence of interventricular septal in the developing heart and caused cardiac hypertrophy in the adult heart (42, 48). *Hand2* overexpression also enhanced cardiomyocyte production by increasing SHF-derived progenitor cell proliferation, which resulted in an enlarged heart with an increased size of outflow tract in the zebrafish model (49). Endocardial cell–specific enhancement of HAND2 within *Nfatc1-Cre* did not show any obvious cardiac phenotypes but improved cardiac ventricular trabeculation at early stage (24). Recently, heterozygous sequence variants in HAND2 have been reported in some patients with congenital heart defects, such as tetralogy of Fallot, patent ductus arteriosus, pulmonary atresia, pulmonary stenosis, atrial or ventricular septal defects, double outlet right ventricle, and dilated cardiomyopathy (50–53).

We observed that the expression of HAND2 in cardiac endothelium is selectively located in endocardial ECs using a HAND2-specific antibody generated recently (46), suggesting a potential role of *Hand2* in the Endo in the regulation of coronary vessel formation. Previous studies have shown that valve phenotypes in *Hand2*-null and *Hand2; Nfatc1-Cre*–deleted mice were not observed in our *Hand2* endocardial deletion mice, indicating that HAND2 may regulate valve formation before coronary angiogenesis initiation (24, 34). Meanwhile, apart from the well-characterized functions of HAND2 in trabeculae and valve formation, our study identified a different role of *Hand2* in regulating coronary artery formation, which parallelly mediated by hypoxic/metabolic changes and TGF-β–induced EMT. This finding indicates that endocardial HAND2 timely controls key cardiac developmental events to maintain cardiac structure and function.

Previous studies have reported that Endo and coronary endothelium can compensate for each other during development. Deletion of the SV contributes to ECs by ablating *Aplnr* to block ELA-APJ signaling, which can increase the expansion of Endo-derived coronary endothelium to compensate for the defect in SV-derived vessel formation (54). Recently, Zhang and colleagues illustrated that elimination of endocardial cells leads to expansion of coronary vessels from the SV to compensate for the loss of coronary vessels derived from the Endo (55). Although this phenomenon of reciprocal compensation has been observed at the cellular level, the distinction between these two populations at the molecular level remains unclear. In our study, we found that *Hand2* ablation downregulated the expression of numerous Endo-specific genes and TFs and upregulated the expression of coronary endothelium-enriched genes in the region of Endo, indicating that genes from these two sources inhibit each other at the molecular level to adopt cell fate commitment beyond cell heterogeneity during coronary development.

Our results also demonstrated that HAND2 acts upstream of hypoxic and glycolytic pathways and negatively regulates them. HAND2 directly binds the promoters of key regulators in these pathways to control endothelial metabolism and behavior. In *Hand2-EKO* mice, hypoxic pathway upregulation increased endocardial sprouting tip cells, but these cells remained confined to the Endo and failed to invade the intramyocardial wall to form arterial ECs. These might be attributable in part to Endo-MT and ECM defects caused by HAND2-mediated TGF-β and p38MAPK alteration.

In summary, our results illustrated the molecular regulatory mechanism of Endo for coronary angiogenesis and arterialization and identified an essential endocardial TF, *Hand2*, which controls the dynamics of endocardial ECs and coronary artery formation by coordinating the GRN, and may be a potential therapeutic target to promote neovasculogenesis in patients with myocardial infarction through reprogramming of the regulatory network for the treatment of coronary artery disease.

## Methods

Additional methods are described in Supplemental Methods.

### Sex as a biological variable.

Our study analyzed embryonic hearts from both male and female mice and showed no significant sexual differences. Accordingly, sex was not regarded as a biological variable in this research.

### Mice.

The mouse lines used in this study are as follows: *Hand2^fl/fl^* (44), *Gata4^fl/fl^* (generated by Shanghai Biomodel Organism Co. Ltd.), *Hif1α^fl/fl^* (56), *Hif2α^fl/fl^* (57), *Vhl^fl/fl^* (58), *Nfatc1-cre* (11), *Bmx-creERT2* (59), *Pdgfb-iCre-ERT2-IresEGFP* (60), *PAC-Cdh5-CreERT2* (61), and *iSure-Cre* (28). The strategies for drug treatment are described in Supplemental Methods. Genotyping primers are listed in Supplemental Table 1. Dosing and dissection schedules for individual experiments in Supplemental Table 2.

### Tissue immunostaining.

Embryos were dissected and fixed in 4% paraformaldehyde on ice with shaking for 5–6 hours for whole-mount staining and overnight for sectional analysis. Briefly, for coronary vessel whole-mount IF, hearts were divided into dorsal and ventral parts and trimmed to remove trabeculae after washing with PBS at 4°C. Samples were blocked at room temperature for 2 hours with shaking in blocking solution (10% donkey serum in 0.3% PBST for coronary veins; 10% donkey serum in 1% PBST for coronary arteries), followed by overnight incubation with blocking solution-diluted primary antibodies at 4°C. The next day, after washing with 0.3% PBST, the samples were incubated with secondary antibodies at 4°C overnight. The samples were then washed and mounted in Fluoromount-G (SouthernBiotech, 0100-01) and stored at 4°C until imaging. For sectional IF, hearts were fixed in 4% paraformaldehyde at 4°C for 1 hour before being saturated overnight at 4°C in PBS with 30% sucrose. Samples were then embedded in OCT (Sakura, 4583) and stored at −80°C. Cryosections within 12–14 μm were made for further staining.

After washing, sections were blocked with 10% donkey serum in PBS for 1 hour at room temperature and then incubated with diluted primary antibodies overnight at 4°C. After PBS washes, sections were incubated with secondary antibodies at room temperature for 2 hours. Then, samples were mounted with Fluoromount-G and stored at 4°C until imaging. For histological analysis, paraffin sections were prepared following standard immunostaining protocols above. A Zeiss LSM-900 confocal microscope was used for the imaging. The antibodies used are listed in Supplemental Table 3.

### EdU incorporation assay.

EdU (Invitrogen, A10044) (50 μg/g) was i.p. injected into pregnant females, 1 hour before dissection. Embryonic hearts were isolated for cryosections analysis. The Click-iT EdU reaction cocktails were prepared following the Click-iT EdU Kit’s instructions (Invitrogen, C10340 or C10337). Samples were incubated for 40 minutes, followed by counterstaining with DAPI after labeling with all other primary and secondary antibodies.

### EC isolation, RNA extraction, and qPCR.

For bioinformatics analysis, endocardial and coronary ECs were isolated as previously described (27). In brief, E14.5 *Npr3-CreER; Ai47* hearts (tamoxifen administered at E10.5) and E14.5 *Fabp4-Cre; Ai47* hearts were harvested. Ventricles were collected, minced finely, and then subjected to digestion. GFP^+^ cells were sorted using a FACSAria III flow cytometer (BD Biosciences).

For endothelial/endocardial cells isolation from *Hand2-EKO; iSure-Cre* mice, EC collection was followed by standard protocols as described previously (62). Briefly, cardiac tissues were digested, and dissociated ECs were harvested. The cell pellet was resuspended in blocking solution (5% FBS in Ca²^+^/Mg²^+^-free DPBS) and incubated at 4°C for 20 minutes before incubation with specific antibodies at 4°C for 30 minutes: anti-CD31 antibody (BD Pharmingen, 551262, 1:200) for cardiac ECs and anti-EMCN antibody (Thermo Fisher Scientific, 50-5851-82,1:200) for endocardial ECs. Then, cells were washed and stained with DAPI. For each group, approximately 9,000–12,000 DAPI^–^ APC-CD31^+^/Tomato^+^ cardiac ECs or APC-EMCN^+^/Tomato^+^ endocardial ECs were sorted by a FACSAria III flow cytometer (BD Biosciences). Cells were sorted directly into buffer RLT (RNAeasy Micro kit, Qiagen 74004), and RNA was extracted according to the manufacturer’s instructions and stored at –80°C.

For quantitative real-time PCR (qPCR), total RNA was retrotranscribed with the HiScript III RT SuperMix Kit (Vazyme, R323-01), and qPCR reactions were performed with SYBR qPCR Master Mix (Vazyme, Q312-02). All primer sequences are provided in Supplemental Table 4.

### Western blot analysis.

Embryonic hearts were harvested from timed-pregnant mice based on mouse genotypes and snap-frozen in liquid nitrogen. Tissues and cultured ECs were lysed in ice-cold RIPA lysis buffer (Beyotime, P0013B) supplemented with PhosSTOP (Roche, 4693132001) and phosphatase inhibitor cocktail A (Beyotime, P1081) using a benchtop homogenizer (Eppendorf, BULLET BLENDER). After centrifugation at 12,800 × *g* for 10 minutes at 4°C, supernatants were mixed with 4× loading buffer and heated at 99°C for 15 minutes. Proteins were separated by 10% SDS–PAGE and detected using the antibodies listed in Supplemental Table 3.

### Bulk and scRNA-seq.

For bulk RNA-seq, total RNA was extracted from sorted E14.5 ECs of *Cdh5-CreERT2; iSure-Cre* and *Hand2-EKO; iSure-Cre* mouse hearts, followed by library preparation according to the standard Illumina instructions. The concentration and size distribution of the cDNA library were assessed using an Agilent 4200 bioanalyzer before sequencing with the Illumina NovaSeq 6000 platform. The raw reads were filtered by Seqtk before mapping to genome GRCm38 (mm10) using Hisat2 (version:2.0.4). Gene fragment quantification was performed using StringTie v1.3.3b, with subsequent normalization using the TMM (trimmed mean of M values) method. Transcript expression levels were quantified as fragments per kilobase of exon per million mapped reads, and sequencing, together with GO term enrichment and KEGG pathway analyses, was conducted by Shanghai Biotechnology Corporation.

For scRNA-seq, cardiac ECs were sorted from *Cdh5-CreERT2; iSure-Cre* and *Hand2-EKO; iSure-Cre* mouse E14.5 hearts. A total of 11,000 single cardiac ECs were analyzed per sample using a 10x Genomics Chromium single-cell controller. Single-cell cDNA libraries were constructed using the Single Cell 3’ Library and Gel Bead Kit v3.1, and sequencing was performed on an Illumina NovaSeq 6000 platform. Details for quality control and systematic analysis are presented in Supplemental Methods. The well-known gene markers for cluster annotation are list in Supplemental Table 5.

### CUT&Tag assay.

E14.5 isolated ECs from control and *Hand2-EKO* mice were performed for CUT&Tag assay using a Hyperactive Universal CUT&Tag Assay Kit for Illumina Pro (Vazyme, TD904). Briefly, the ECs were incubated with ConA Beads Pro, resuspended with precooled primary HAND2 antibody (Abcam, ab200040, 1:20) or control IgG, and incubated overnight at 4°C. Samples were then incubated with a secondary antibody (Vazyme, AB207-01) and Hyperactivep A/G-TransposonPro reagent, followed by resuspension in Trueprep Tagment Buffer L and incubation at 37°C for 1 hour. *E. coli* DNA was spiked in for optional normalization. DNA fragments were purified, and libraries were prepared following the manufacturer’s instructions. Then, libraries were sequenced on the Illumina platform (HaploX) for further analysis. Detailed procedures are available in the Supplemental Methods.

### Ventricular explant culture and image.

The ventricular explant assay was as previously described (13). Left ventricles were isolated from E12.0 hearts of *Hand2-EKOBmx; iSure-Cre* or *Bmx-CreERT2; iSure-Cre* embryos and placed in a 4-well dish, which was preloaded with 150 μL Matrigel medium (1:1 volume ratio of basal medium [Gibco, C11995500BT] and Matrigel [Corning, 354230]). 200 μL basal medium was added at day 0. Subsequently, the tissues were treated with 10 ng/mL VEGF165 (PeproTech, 100-20-10ug) in the basal medium for angiogenesis stimulation. Images were captured on days 3 and 9 using a confocal microscope (ZEISS LSM 900) to detect TdTomato^+^ cells.

### Statistics.

Confocal images were quantified using FIJI, and statistical analyses were performed using GraphPad Prism 9. As indicated in the figure legends, two groups of samples were compared using paired or unpaired 2-tailed Student’s *t* tests, as appropriate. Multiple comparisons involving independent groups were analyzed using multiple unpaired Welch’s 2-tailed *t* tests with Benjamini-Krieger-Yekutieli FDR correction. Comparisons among more than two groups were performed using 1-way or 2-way ANOVA followed by Tukey’s multiple-comparison test, as appropriate. Data are presented as mean ± SEM unless otherwise indicated. Differences were considered statistically significant at *P* < 0.05.

### Study approval.

All mouse experiments were performed according to the guidelines of Nanjing University and approved by the Institutional Animal Care and Use Committee of GemPharmatech Co. Ltd in Nanjing, Jiangsu Province (IACUC no. GPTAP002).

### Data availability.

Data supporting the study’s findings are available in this article and can be obtained from the corresponding authors upon reasonable request. Additionally, the RNA-seq, scRNA-seq, and CUT&Tag-seq data reported herein have been deposited in the Gene Expression Omnibus (GEO) database under accession GSE311984, which consists of 3 independent subdatasets: GSE311981 (CUT&Tag), GSE311982 (RNA-seq), and GSE311983 (scRNA-seq). The code used for scRNA-seq data analysis is available at GitHub (https://github.com/Hayesss/endo_whj_scRNA/commit/c3164b5). CUT&Tag data processing and analysis were performed according to the published CUT&Tag workflow (https://yezhengstat.github.io/CUTTag_tutorial/). Values for all graph data points are included in the Supporting Data Values file. Any additional data supporting the study’s findings are available from the corresponding author upon reasonable request.

## Author contribution

W Luo and HW designed experiments, interpreted results, assembled figures, and wrote the manuscript. HW executed the vast majority of wet lab experiments. HZ and HW analyzed bulk RNA-seq, scRNA-seq, and CUT&Tag-seq analysis. LZ, PZ, YW, SD, and W Liu provided general technical assistance and analyzed data. ZY generated and provided essential reagents, antibodies, and mice. W Luo, YC, and ZY discussed the project.

## Conflict of interest

The authors have declared that no conflict of interest exists.

## Funding support

- IMB of Nanjing University start-up funding to Wen Luo.
- National Natural Science Foundation of China Outstanding Young Scientists Fund (Overseas) to W Luo.
- Jiangsu Province Distinguished Professor fund to W Luo.
- National Natural Science Foundation of China (32270872 to W Luo, 92468103 to ZY).

## Supplementary Material

Supplemental data

Unedited blot and gel images

Supporting data values

## Figures and Tables

**Figure 1:**
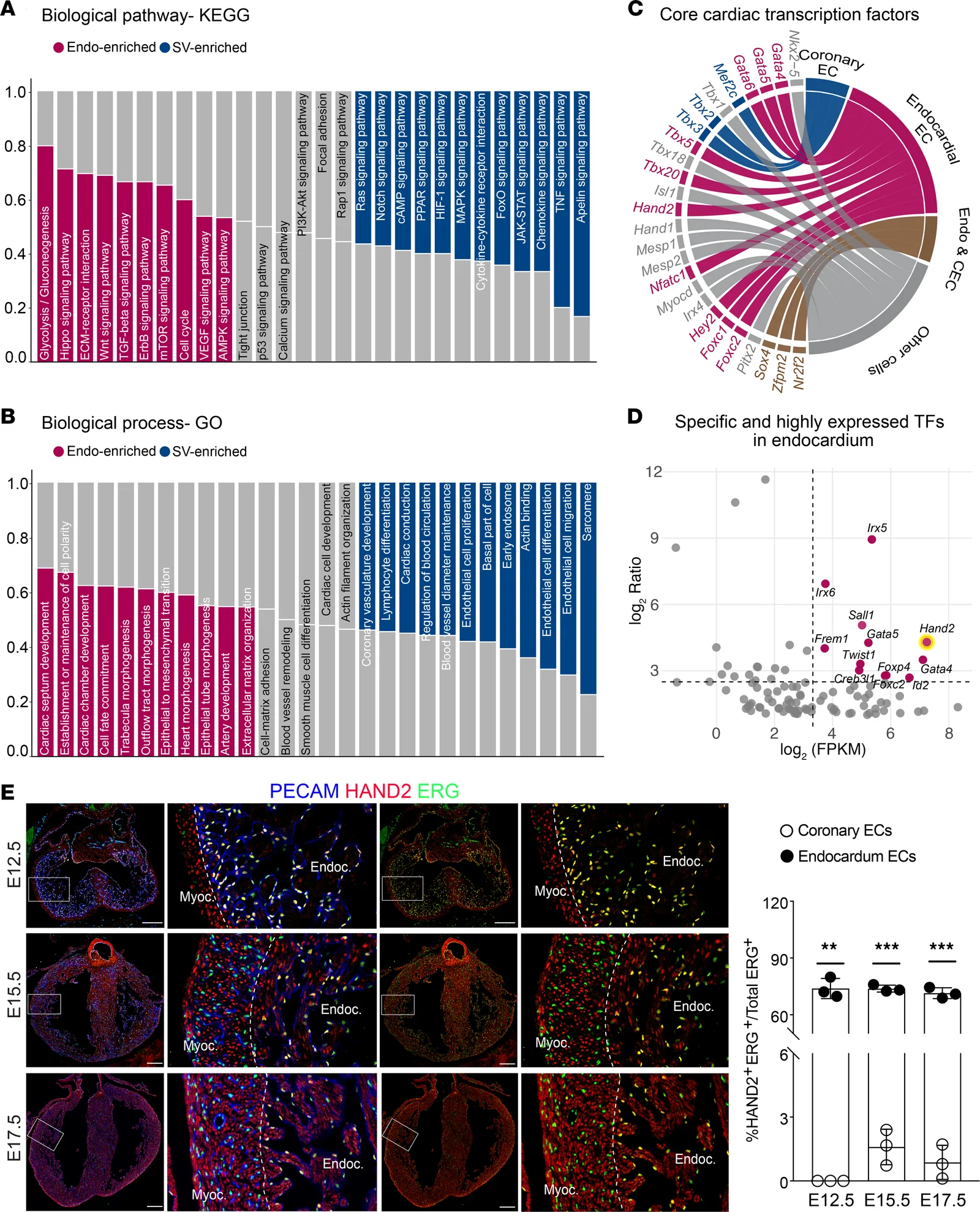
Transcriptome analysis of coronary endothelium identified endocardial enriched transcription factor. (**A**) Kyoto Encyclopedia of Genes and Genomes (KEGG) analysis of SV-enriched and Endo-enriched ECs from E14.5 hearts revealed different signaling pathways. (**B**) Gene Ontology (GO) enrichment analysis of endocardium and coronary ECs showing different functional categories. (**C**) Circular plot illustrating the expression patterns of core cardiac transcription factors across distinct endothelial cell populations. (**D**) Scatter plot showing the expression level [*x* axis, log_2_(FPKM)] and tissue specificity (*y* axis, log_2_ ratio) of transcription factors in the endocardium. (**E**) Immunofluorescence staining of HAND2 and ERG showing specific expression of HAND2 in endocardium from E12.5 to E17.5. Original magnification ×5 (upper), ×6.3 (middle), ×6.8 (lower). Boxed regions are shown at higher magnification to the right. *n* = 3 hearts per group. Data represent mean ± SEM. Statistical significance was assessed using 2-tailed paired *t* tests with Benjamini-Krieger-Yekutieli FDR correction. **P < 0.01, ***P < 0.0001. Endoc., endocardium; Myoc., myocardium. Scale bars: 200 μm.

**Figure 2:**
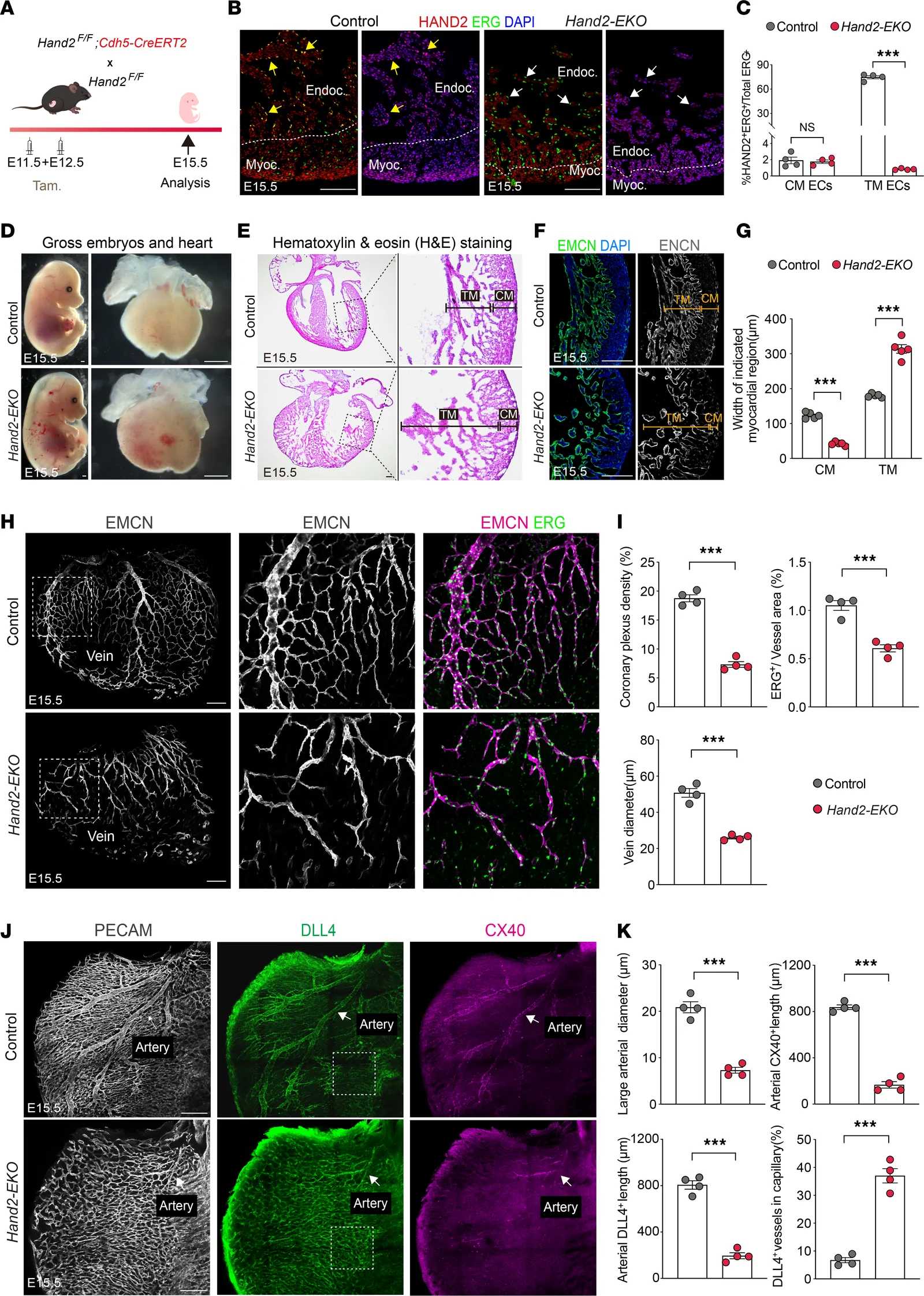
*Hand2* is required for normal cardiovascular development. (**A**) Experimental strategy for *Hand2* deletion in cardiac endothelium. (**B**) Costaining of HAND2 and ERG of E15.5 WT and *Hand2-EKO* sections showing *Hand2* deletion efficiency. The yellow arrows indicate ERG^+^HAND2^+^ ECs in the endocardium in controls; the white arrows represent ERG^+^HAND2^–^ ECs in the endocardium in *Hand2-EKO* mice, which demonstrate the gene deletion efficiency. (**C**) Quantification of HAND2^+^ERG^+^ cells in compact myocardium ECs (CM ECs) and trabecular myocardium ECs (TM ECs). *n* = 4 hearts per group. (**D**) Bright-field images of E15.5 gross embryos and hearts showing defective embryogenesis. (**E**) H&E staining showing marked thinning of ventricular myocardial wall and hypertrabeculation in *Hand2-EKO* hearts at E15.5. *n* = 3 hearts per group. (**F** and **G**) Immunostaining of EMCN showing decreased myocardial thickness in E15.5 *Hand2-EKO* hearts and (**G**) quantifications. *n* = 5 hearts per group. (**H** and **I**) Whole-mount staining of E15.5 coronary veins showing decreased density, diameter of veins, and ERG^+^ cell numbers with (**I**) quantification analysis. *n* = 4 hearts per group. (**J** and **K**) Confocal images showing absence of normal coronary artery formation and extension in E15.5 *Hand2-EKO* hearts and (**K**) quantifications. *n* = 4 hearts per group. Data represent mean ± SEM. Statistical significance was assessed using multiple unpaired 2-tailed *t* tests with Benjamini-Krieger-Yekutieli FDR correction for (**C**) and (**G**) and unpaired 2-tailed Student’s *t* tests for (**I** and **K**). ***P < 0.0001. Scale bars: 200 μm (**B**, **E**, **F**, **H** and **J**); 500 μm (**D**).

**Figure 3:**
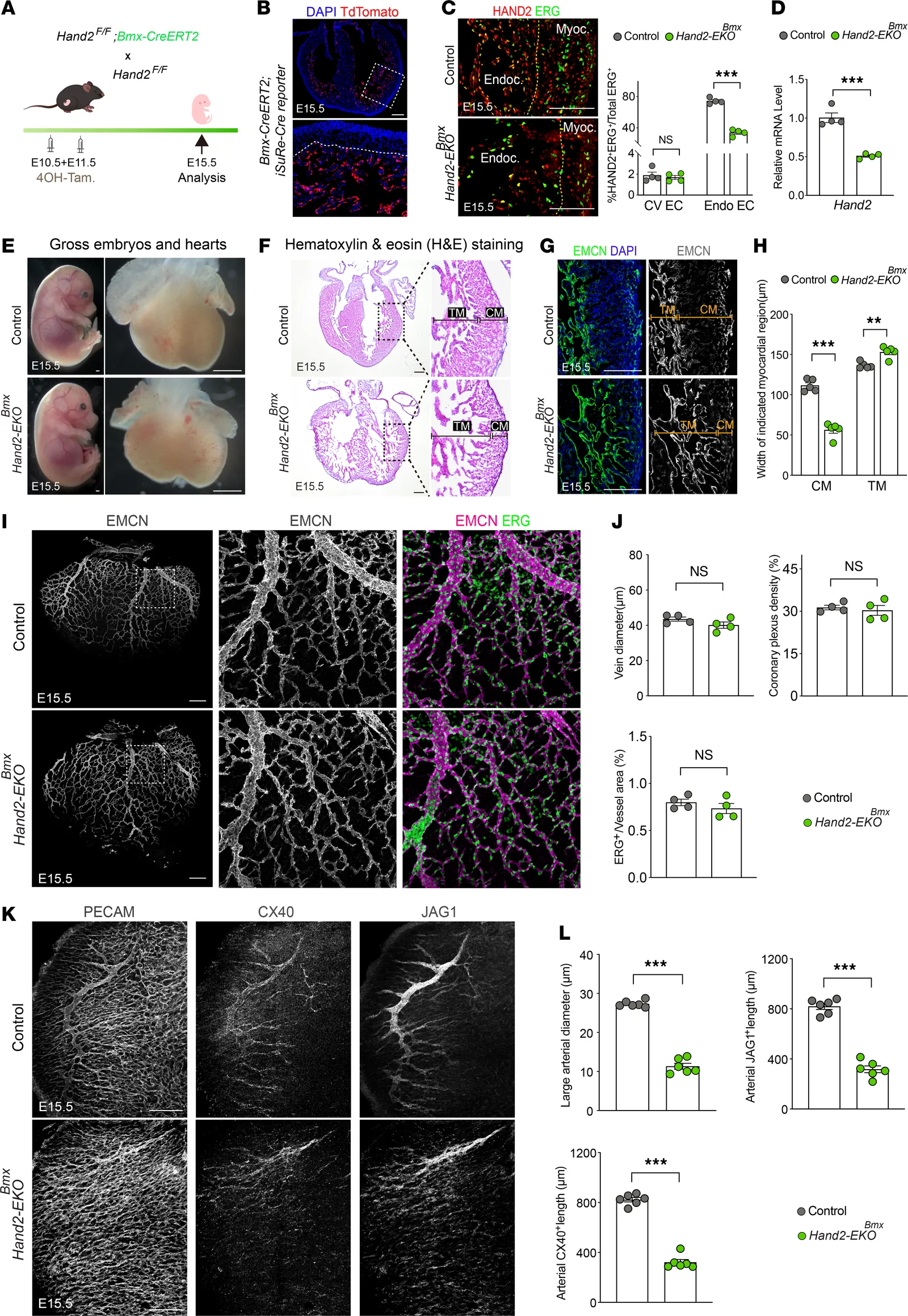
Endocardial deletion of *Hand2* caused defective cardiovascular development. (**A** and **B**) Experimental strategy of *Hand2* ablation in endocardium with *Bmx-creERT2* lineage and Cre expression pattern in *iSuRe-Cre; Bmx-creERT2* mice. (**C**) Confocal images showing deletion of *Hand2* in endocardium of E15.5 *Hand2-EKO*^Bmx^ hearts with quantification analysis. *n* = 4 hearts per group. (**D**) qPCR analysis of *Hand2* mRNA level in *Hand2-EKO^Bmx^* heart ECs. *n* = 4 samples per group. (**E**) Bright-field images of E15.5 gross embryos and hearts showing abnormal embryonic and cardiac morphologies. (**F**) H&E staining showing decreased myocardial wall thickness in *Hand2-EKO^Bmx^* hearts at E15.5. *n* = 3 hearts per group. (**G** and **H**) Representative images (**G**) and quantitative analysis (**H**) of EMCN^+^ endocardium indicate hypertrabeculation in *Hand2-EKO^Bmx^* hearts at E15.5. *n* = 5 hearts per group. (**I** and **J**) Whole-mount staining of EMCN and ERG in coronary veins showing normal venous plexuses formation with quantification analysis. *n* = 4 hearts per group. (**K** and **L**) Whole-mount staining of arterial markers showing abnormal coronary artery development and remodeling in *Hand2* mutants, with decreased diameter and length of arteries (**K**), and quantification analysis (**L**). *n* = 6 hearts per group. Data represent mean ± SEM. Statistical significance was assessed using multiple unpaired 2-tailed *t* tests with Benjamini-Krieger-Yekutieli FDR correction for (**C** and **H**) and unpaired 2-tailed Student’s t tests for (**D**, **J**, and **L**). **P < 0.01, ***P < 0.001. Scale bars: 200 μm (**B**, **F**, **G**, **I** and **K**); 100 μm (**C**); 500 μm (**E**).

**Figure 4:**
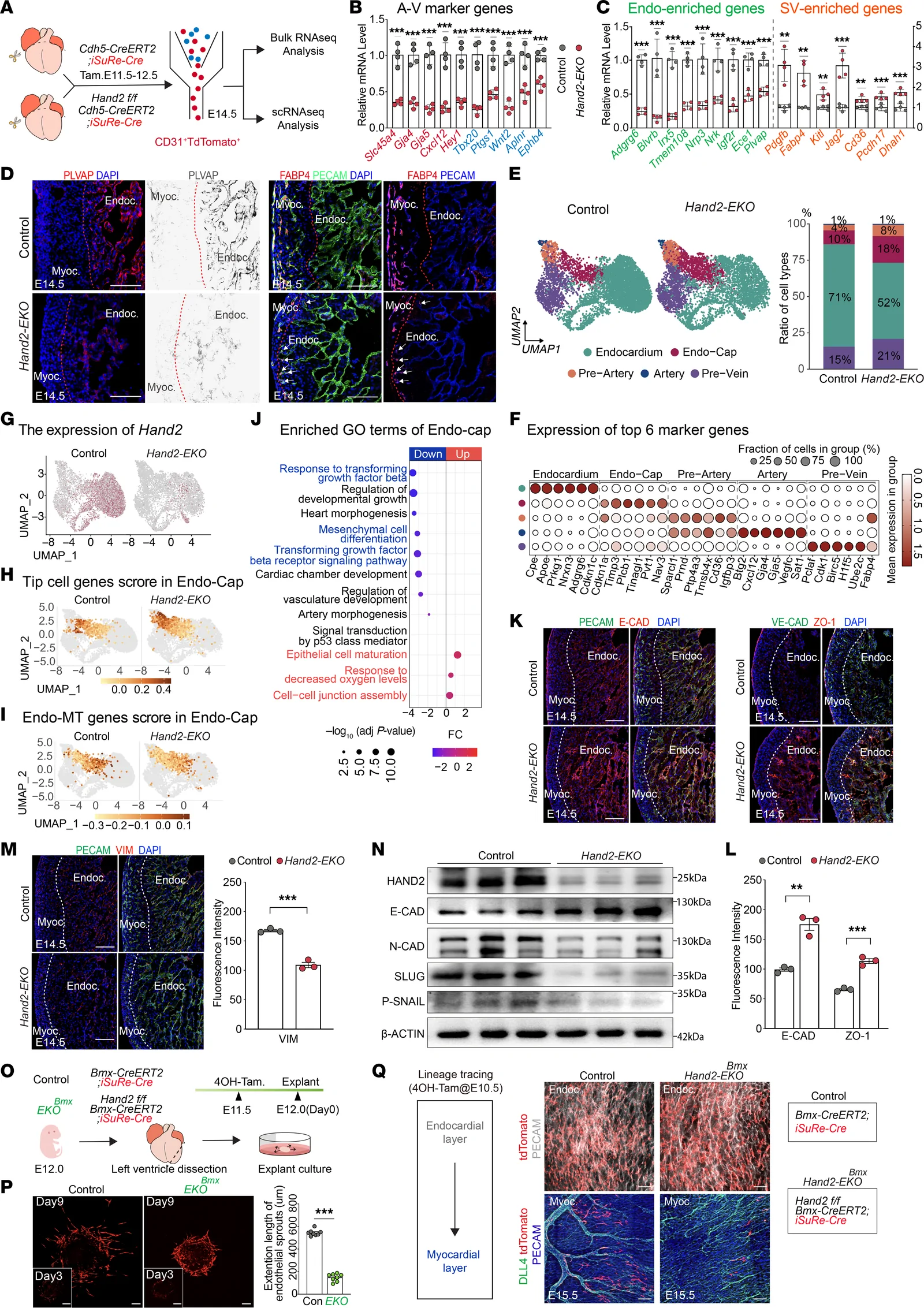
Bulk and single-cell RNA-Seq analysis in *Hand2-EKO* hearts revealed decreased coronary angiogenesis. (**A**) Schematic illustration of strategies for tamoxifen injection and EC preparation for bulk and single-cell RNA-seq. (**B**) qPCR analysis of arterial marker genes and venous marker genes. *n* = 4 samples per group. (**C**) qPCR analysis of SV-enriched and Endo-enriched genes. *n* = 4 samples per group. (**D**) Confocal images showing decreased expression of endocardial marker PLVAP and ectopic increased SV marker FABP4 in *Hand2-EKO* endocardium. *n* = 3 hearts per group. (**E**) UMAP representation of the ECs of control and *Hand2-EKO* hearts. Bar graph showing each cluster proportion in the ECs of control and *Hand2-EKO* hearts. (**F**) Dot plot showing the top 6 marker genes of subclusters. (**G**) The expression of *Hand2* in the ECs of control and *Hand2-EKO* hearts. (**H** and **I**) Tip cell (**H**) and Endo-MT (**I**) module scores in Endo-Cap cell cluster. (**J**) Enriched GO terms of Endo-Cap differentially expressed genes. (**K**–**M**) Immunofluorescence staining demonstrates decreased Endo-MT process in *Hand2* mutants. *n* = 3 hearts per group. (**N**) Western blotting analysis of Endo-MT associated markers. *n* = 3 samples per group. (**O**) Schematic showing Matrigel coronary angiogenesis assays using E12.0 left ventricular explant of *Hand2-EKO^Bmx^* and control mice. (**P**) Representative images and quantitative analysis of EC migration. *n* = 8 hearts per group. (**Q**) Whole-mount immunostaining of E15.5 hearts for tdTomato, PECAM, and DLL4 showing migration of endocardial cells, labeled by induction at E10.5, from the endocardial layer into the myocardial layer. *n* = 3 hearts per group. Data represent mean ± SEM. Statistical significance was assessed using an unpaired 2-tailed Student’s *t* test (**B**, **C**, **L**, **M** and **P**). **P < 0.01, ***P < 0.001. Scale bars: 100 μm (**D**, **K**, **M**, **P** and **Q**). Endo-Cap, endocardial-derived capillaries; Pre-Vein, prevenous cells.

**Figure 5:**
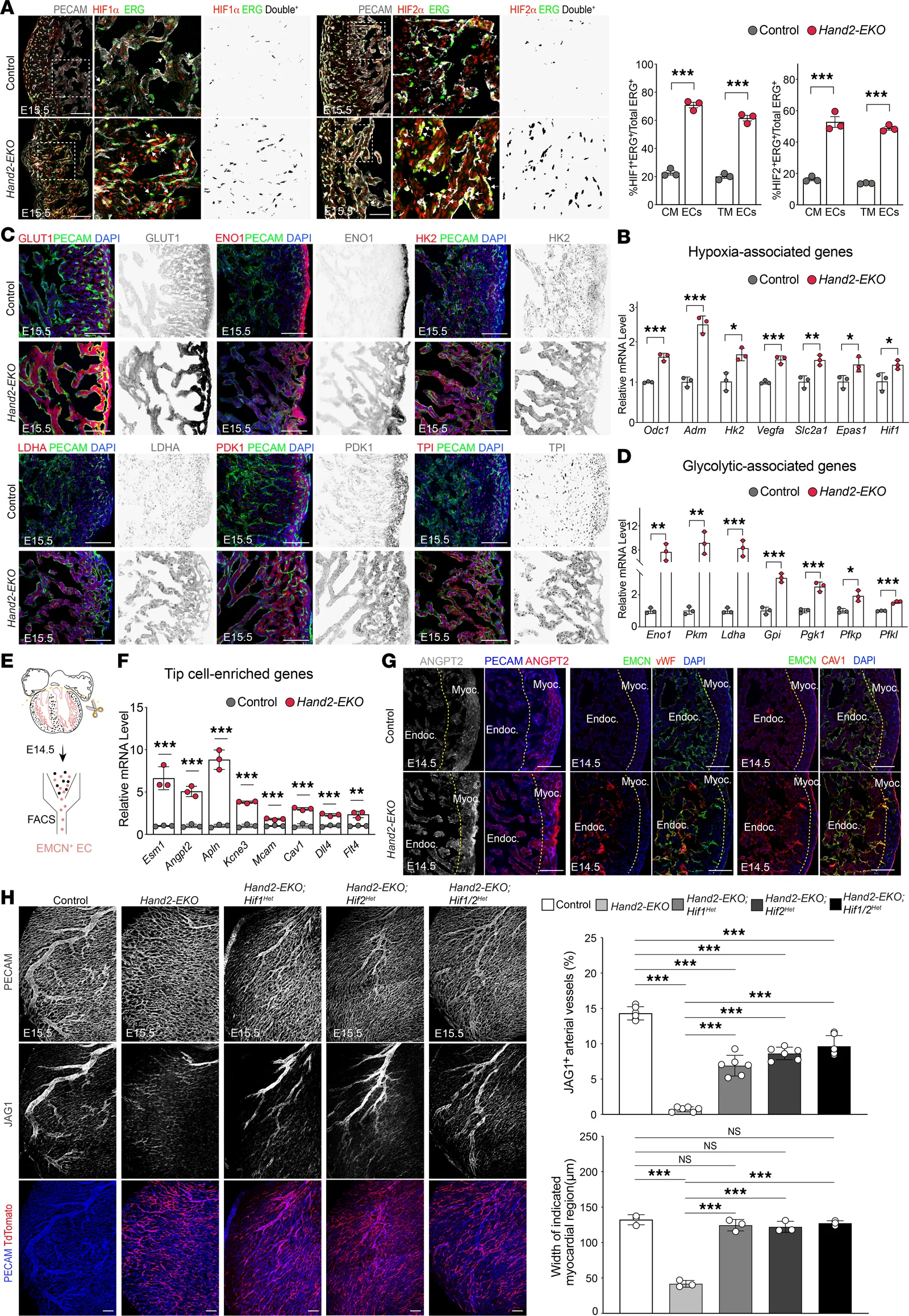
*Hand2* deficiency in endothelium elevated hypoxia signaling and enhanced tip cell activity. (**A**) Representative images and quantitative analysis of hypoxia response factors HIF1α and HIF2α in E15.5 *Hand2-EKO* hearts. *n* = 3 hearts per group. (**B**) qPCR analysis of hypoxia-associated genes from ECs of E15.5 *Hand2-EKO* hearts. *n* = 3 samples per group. (**C**) Confocal images showing upregulated downstream targets, including GLUT1, ENO1, HK2, LDHA, PDK1, and TPI in *Hand2* mutants. *n* = 3 hearts per group. (**D**) qPCR analysis of glycolysis-associated genes from ECs of E15.5 *Hand2-EKO* hearts. *n* = 3 samples per group. (**E**) Schematic illustration of strategies of EMCN^+^ endocardial cells sorting for qPCR analysis. (**F**) qPCR analysis of tip cell–enriched genes from EMCN^+^ ECs of E14.5 hearts. *n* = 3 samples per group. (**G**) Immunostaining of tip cell markers, such as ANGPT2, vWF, and CAV1, showing increased expression in endocardium. *n* = 3 hearts per group. (**H**) Whole-mount staining showing the partial rescue in coronary arteries after suppressing Hif pathway in *Hand2-EKO* hearts with quantification analysis. *n* = 6 hearts per group. The width of myocardial region and quantification analysis of JAG1^+^ arterial vessels of each group. *n* = 3 hearts per group. Data represent mean ± SEM. Statistical significance was assessed using an unpaired 2-tailed Student’s *t* test (**A**, **B**, **D** and **F**) or 1-way ANOVA (**H**) followed by Tukey’s multiple-comparison test. *P < 0.05, **P < 0.01, ***P < 0.001. Scale bars: 100 μm (**A**, **C**, **G** and **H**).

**Figure 6:**
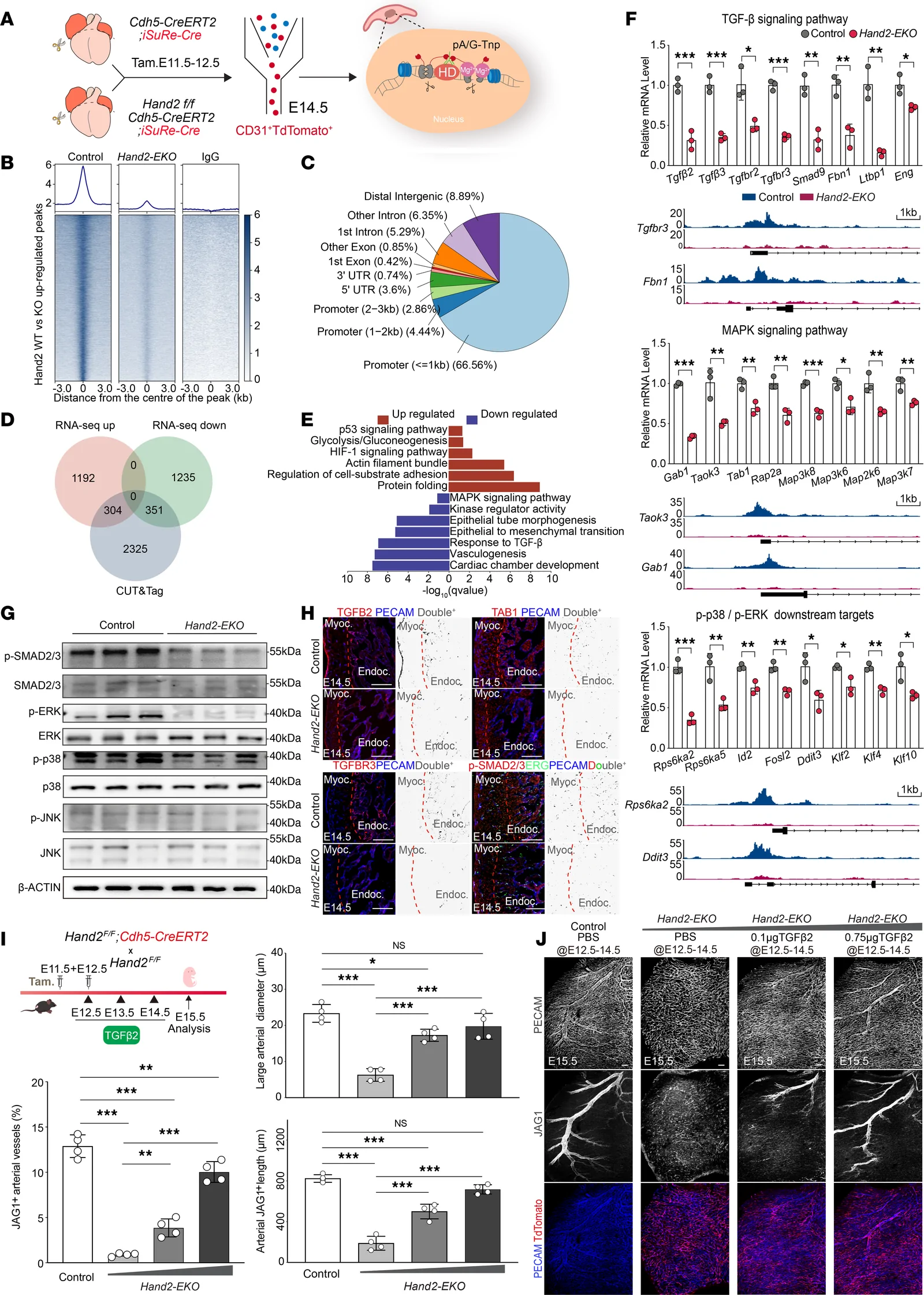
*Hand2* coordinated TGF-β MAPK/p38 pathways control EC migration and artery formation. (**A**) Schematic illustration of strategies for EC collection and CUT&Tag assay. (**B**) Heatmap showing decreased binding of HAND2 target genes in *Hand2-EKO* mice. (**C**) Annotation of HAND2 binding peaks in endothelial cells. (**D**) Intersection plot showing the overlap of RNA-seq and CUT&Tag data analysis. (**E**) GO-KEGG analysis of gene signatures from overlapped genes. (**F**) qPCR analysis and genome browser tracks of CUT&Tag signals at the *Tgfbr3*, *Fbn1*, *Taok3*, *Gab1*, *Rps6ka2*, and *Ddit3* loci. *n* = 3 samples per group. (**G**) Western blotting analysis of TGF-β target proteins, including SMAD2/3, p-SMAD2/3, ERK, p-ERK, p38, p-p38, JNK, and p-JNK, in control and *Hand2* mutant hearts. *n* = 3 samples per group. (**H**) Immunostaining of TGF-β2, TAB1, TGF-BR3, and p-SAMD2/3 showing decreased TGF-β signaling in *Hand2-EKO* hearts. *n* = 3 hearts per group. (**I** and **J**) Schematic illustration of strategies for tamoxifen and TGF-β2 recombinant protein injection. Confocal images showing restored coronary artery formation by treatment with TGF-β2 recombinant protein in E15.5 *Hand2-EKO* hearts and quantification analysis. *n* = 4 hearts per group. Data represent mean ± SEM. Statistical significance was assessed using an unpaired 2-tailed Student’s *t* test (**F**) or 1-way ANOVA (**I**) followed by Tukey’s multiple-comparison test. *P < 0.05, **P < 0.01, ***P < 0.001. Scale bars: 100 μm (**H** and **J**).

**Figure 7:**
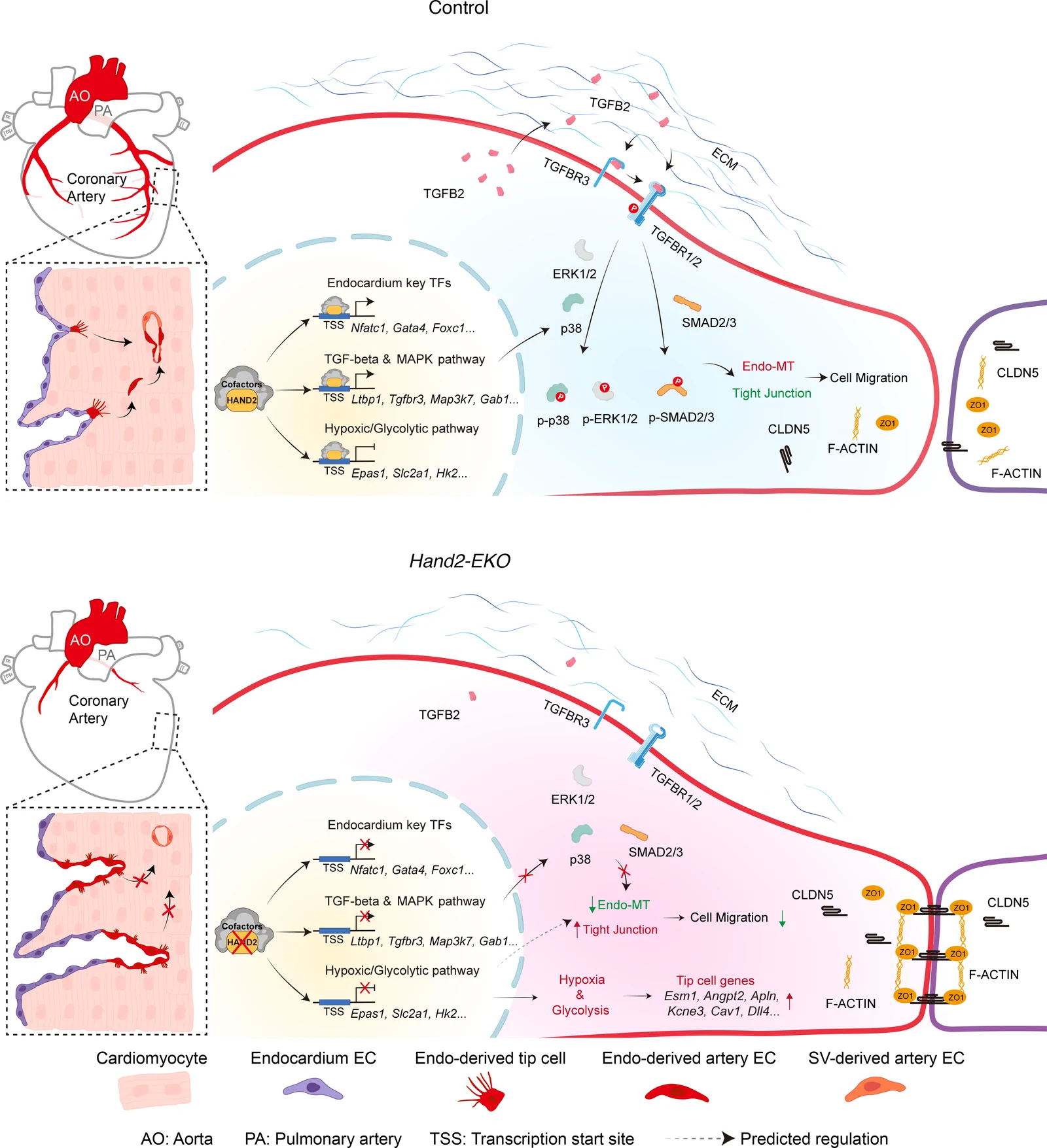
Proposed transcriptional regulatory mechanisms of *Hand2* for coronary artery development. Endocardium is a major contributor to coronary arteries during early heart development, which is spatiotemporally regulated by multiple signaling pathways that sequentially control endocardial angiogenesis (Endo-MT) and endocardial-to-coronary vessel fate conversion. *Hand2* functions as an essential upstream transcription factor that coordinates the endocardial gene regulatory network (GRN) in the developing heart. HAND2 binds to gene promoters to modulate signaling for normal cardiovascular development (top). Endocardial loss of *Hand2* disrupts this complex transcriptional orchestration, impairs TGF-β–SMAD2/3 and p38 MAPK–mediated Endo-MT, upregulates hypoxia-induced glycolysis and tip cell-enriched gene expression, and eventually results in defective coronary artery formation (bottom). AO, aorta; PA, pulmonary artery; TSS, transcription start site.

## Version 1 08/06/2026

In-Press Preview

## Version 2 10/01/2026

Electronic publication
