# Supplementary material for: Endocardial transcription factor HAND2 orchestrates hypoxic and TGF-β signaling to modulate coronary artery formation
Source: J Clin Invest. 2026 Aug 6;136(19):e203310. doi: 10.1172/JCI203310 (PMC13626820; doi:10.1172/JCI203310)
Supplement: Supplementary file 1 [file jci-136-203310-s141.pdf]

Endocardial transcription factor HAND2 orchestrates hypoxic and TGF-β signaling to modulate coronary artery formation

Table of contents:

Supplemental Materials and Methods..2

Supplemental Figure Legend..11

Supplemental references...27

Supplemental Materials and Methods

Drug administration and induction in mice

For *Pdgfb-iCre-ERT2-IresEGFP* and *PAC-Cdh5-CreERT2* inductions, pregnant females were orally gavaged with 200 μL of corn oil containing tamoxifen (20 mg/mL; Sigma-Aldrich, T5648) and progesterone (10 mg/mL; Sigma-Aldrich, P0130) at the indicated embryonic stages. For *Bmx-creERT2* induction, 200 μL of corn oil containing 4-OH tamoxifen (7.5 mg/ml; Sigma-Aldrich, H6278) and progesterone (7.5 mg/ml) was administered to pregnant females by oral gavage at the indicated stage. Stages of injection and dissection for each embryo are listed in Supplemental Table 2.

For pharmaceutical administration of TGFβ2 recombinant protein, exogenous TGFβ2 at doses of 0.1 μg and 0.75 μg was injected intraperitoneally into pregnant *Hand2-EKO* mice from E12.5 to E14.5, one time per day. Negative controls (Wild type) and positive controls (*Hand2-EKO*) were treated with PBS. Hearts were harvested at E15.5 and processed for whole-mount staining (1, 2).

Immunohistochemistry

Cryosections within 12–14 μm were made for immunohistochemistry (IHC). After washing, sections were performed using the HRP-DAB IHC Detection Kit (Starter, S0C1001) following the manufacturer's instructions. After primary antibody incubation, sections were washed with PBS (3×5 min), and incubated with HRP-conjugated secondary antibody at RT for 1 h. Developed with DAB substrate for 5–10 min, the reaction was stopped with distilled water. Sections were mounted with Fluoromount-G

and stored at 4°C until imaging. An Olympus BX43 microscope was used for the imaging. The antibodies used are listed in Supplemental Table 3.

Single-cell RNA sequencing analysis

Cell quality was assessed based on the number of detected genes, mitochondrial read fraction, doublet score, and estimated ambient RNA contamination. Cells were retained if they contained more than 400 detected genes and had mitochondrial reads accounting for less than 10% of total reads. Genes detected in fewer than three cells per group were excluded. Doublets were identified using a modified DoubletFinder-based approach (3). and removed when doublet scores exceeded 0.3. Ambient RNA contamination was estimated using DecontX (4), and cells with contamination scores ≥0.5 were excluded. After quality control filtering, 6,019 *Hand2-EKO; iSure-Cre* endothelial cells and 7,063 *Cdh5-CreERT2; iSure-Cre* control endothelial cells were retained for downstream analyses. A small number of lymphatic endothelial cells and valve endothelial cells were excluded before downstream cardiac endothelial analyses.

Dimensionality reduction, clustering, and annotation

Dimensionality reduction and clustering were performed using Seurat. Principal component analysis was performed on the scaled expression matrix using RunPCA with 50 principal components (5). The first 30 principal components were retained for downstream analyses. Cells were embedded into a two-dimensional space using UMAP. A K-nearest-neighbor graph was constructed using Euclidean distances in PCA space with k = 30, and cell clusters were identified using the Louvain algorithm implemented in Seurat's FindNeighbors and FindClusters functions at a resolution of 0.8.

Cluster identities were assigned based on canonical endothelial marker genes, cluster-specific marker genes, and comparison with published cardiac endothelial cell annotations.

Pseudotime ordering and developmental trajectory inference

Pseudotime trajectories were inferred using Slingshot v2.7.0 (6). Manually annotated endothelial populations, including Endocardium, Endo-Cap, Pre-A, Art, and Pre-V, were used as cluster labels. The Endocardium cluster was designated as the root cluster based on prior knowledge of cardiac endothelial development (start.clus = "Endocardium"), and no terminal cluster was pre-specified (end.clus = NULL).

For the primary analysis, trajectories were fitted in the UMAP embedding space (reducedDim = "UMAP"). Slingshot inferred lineage relationships by constructing a minimum spanning tree across endothelial populations, followed by simultaneous principal curve fitting. Principal curves were approximated using 150 equidistant points (approx_points = 150) and smoothed using the lowess algorithm (smoother = "lowess"). Curve fitting was iterated until convergence or until a maximum of 15 iterations was reached (maxit = 15), using a convergence threshold of 0.001.

At branching points, curves were shrunk toward shared trajectories using cosine-distance-based shrinkage (shrink = TRUE, shrink.method = "cosine"). Curves were allowed to extend beyond terminal clusters to accommodate cells near trajectory endpoints (extend = "y", stretch = 2). All cells were allowed to contribute to at least one lineage without outlier exclusion (omega = Inf). Cell weights and lineage assignments

were iteratively updated during curve fitting (reweight = TRUE, reassign = TRUE). A global random seed of 42 was used for reproducibility.

The inferred pseudotime values and lineage assignments were extracted and incorporated into the Seurat object metadata for downstream visualization, group-wise comparison, and analysis of gene expression dynamics along endothelial differentiation trajectories.

To evaluate whether the inferred trajectory was dependent on clustering granularity, Slingshot analysis was repeated using Louvain clusters generated at multiple resolutions, including 0.5, 0.8, 1.0, and 1.5. For each resolution, the cluster with the largest overlap with manually annotated Endocardium cells was used as the root cluster. The resulting pseudotime values were compared with the baseline pseudotime using Spearman correlation across shared cells. Pairwise Spearman correlations were also calculated among trajectories inferred from different clustering resolutions to assess the stability of cell ordering across clustering granularities.

To assess the effect of root-cluster specification and trajectory topology, Slingshot was repeated using alternative starting conditions. Endocardium, Endo-Cap, and Art were independently specified as root clusters, and an unconstrained analysis was also performed without specifying a root cluster. The resulting pseudotime values were compared with both the baseline pseudotime and the predefined endothelial progression order using Spearman correlation. These analyses were used to evaluate the stability of trajectory topology and the dependence of trajectory directionality on biologically informed root assignment.

To test whether the inferred trajectory was sensitive to the choice of dimensionality reduction method, Slingshot was repeated in alternative embedding spaces, including Harmony-corrected PCA and diffusion map coordinates. For each embedding method, the primary endocardial-to-arterial lineage was selected based on its agreement with the predefined Endocardium–Endo-Cap–Pre-A–Art progression order. Pseudotime values inferred from different embedding spaces were compared using pairwise Spearman correlations across cells with non-missing pseudotime values.

To further assess whether the selected trajectory reflected lineage progression rather than a transient transcriptional state transition, the distribution of annotated endothelial populations and representative lineage-associated marker genes was examined along pseudotime. The relative ordering of Endocardium, Endo-Cap, Pre-A, and Art cells was compared with the predefined developmental progression order. In parallel, the expression patterns of endocardial- and arterial-associated markers, including *Dll4*, *Gja5*, *Hey1*, and *Efnb2*, were visualized along pseudotime to assess progressive changes in endothelial identity.

Pseudotime-associated gene analysis

Pseudotime-associated genes were identified using tradeSeq-based generalized additive models along the inferred endothelial differentiation trajectory (7). The associationTest function in tradeSeq was used to evaluate genes associated with pseudotime, and genes with $p < 0.05$ were retained as pseudotime-associated genes. Gene expression dynamics along the differentiation trajectory were visualized using ggplot2.

Differential gene expression analysis

Cluster-specific marker genes were identified using Seurat's FindAllMarkers function with test.use = "MAST" on log-normalized expression data without cell-cycle correction (8). Genes were tested using min.pct = 0.25 and logfc.threshold = 0.25. P-values were adjusted for multiple comparisons using the Benjamini–Hochberg method, and genes with adjusted $p < 0.05$ were considered significantly differentially expressed.

Endothelial signature scoring

Endothelial transcriptional programs were quantified using the UCell method (9) implemented in the irGSEA framework. Curated arterial endothelial, venous endothelial, endocardial endothelial, and tip-cell gene signatures were used as input gene sets (Supplemental Table 5). UCell scores were calculated for each cell based on the relative ranks of signature genes within the single-cell expression profile, thereby reducing the influence of sequencing depth and global expression differences across cells. The resulting scores were added to the single-cell metadata and used for downstream visualization and comparison across endothelial subpopulations, developmental trajectories, and experimental groups.

Re-analysis of E12.0 and E17.5 cardiac endothelial datasets

To assess endothelial transcriptional programs across developmental stages, one E12.0 and one E17.5 mouse cardiac endothelial single-cell RNA-seq dataset from the same study were jointly re-analyzed within a consistent data source (10). These datasets were processed as independent reference or validation datasets rather than as part of the primary E14.5 *Hand2-EKO* analysis.

Raw or processed count matrices were imported into OmicVerse (11) and analyzed using a unified workflow where applicable. Cells from different samples or experimental conditions were merged based on shared genes, while sample identity, developmental stage, and group information were retained in the metadata. Low-quality cells were removed based on gene complexity and mitochondrial read fraction. Unless otherwise specified by the original dataset, cells with fewer than 1,000 detected genes or more than 10% mitochondrial reads were excluded.

Count matrices were normalized to 10,000 counts per cell and log-transformed. Highly variable genes were selected, followed by regression of technical covariates, including total UMI count, detected gene number, mitochondrial fraction, and cell-cycle scores when available. Dimensionality reduction and clustering were performed using PCA, K-nearest-neighbor graph construction, UMAP embedding, and Leiden clustering.

Endothelial transcriptional programs in these additional datasets were scored using the same rank-based UCell strategy described above. The resulting arterial endothelial, venous endothelial, endocardial endothelial, and tip-cell signature scores were added to the cell metadata and used for downstream visualization and comparison across developmental stages and endothelial subpopulations.

CUT&Tag sequencing analysis

Adapters and low-quality bases were trimmed with fastp (v0.23.4). Bowtie2 (v2.5.3) with TF-friendly parameters (--very-sensitive --no-mixed --no-discordant -X 2000) was used to align the reads to the mm10 genome. Alignments with MAPQ<30 and

mitochondrial reads were filtered using SAMtools (v1.21). PCR duplicates were not removed, and library complexity and fragment size distributions were checked. MACS2 (v2.2.7.1) was used for peak calling in narrow-peak mode with the following parameters: callpeak -f BAMPE -q 0.01 --keep-dup all. For each replicate, the corresponding IgG control was used to estimate background enrichment. Signal tracks were generated using deepTools (bamCoverage, bin = 25 bp, normalized by RPGC) for visualization in IGV. Annotation, motif analysis, and differential binding peaks were annotated using ChIPseeker (v1.38.0) against TxDb. Mmusculus. UCSC.mm10. knownGene, assigning the nearest TSS (±50 kb) and classifying promoter (±3 kb), intragenic, and distal sites. Aggregate plots and heatmaps around HAND2 motifs or peak summits were created using deepTools(v3.5.1) (computeMatrix reference-point + plotHeatMap/ plotProfile).

In vitro HUVEC culture and transfection

Human umbilical vein endothelial cells (HUVECs) (PromoCell, c-12208) were cultured in Medium 199 (Gibco) containing 20% (v/v) fetal bovine serum (FBS), 10 IU/mL basic fibroblast growth factor (bFGF/FGF2), and 1% penicillin-streptomycin. The cell culture was maintained under standard conditions at 37°C in a humidified incubator with 5% CO_2.

For transient transfection, HUVECs were seeded into antibiotic-free medium 24 h before treatment. Cells were transfected with either the pLV3-GFP control vector or the pLV3-HAND2 overexpression plasmid (P82357; Wuhan Miaoling Biotechnology Co.,

Ltd.) using Lipofectamine 2000 (11668-019; Invitrogen) according to the manufacturer's instructions. At 24 h post-transfection, cells were harvested for subsequent functional assays.

Tube Formation Assay

To evaluate capillary-like tube formation, 24-well plates were coated with Matrigel (0827015; ABWbio) diluted according to the manufacturer's instructions. The coated plates were incubated at 37°C in a 5% CO_2 for 30 min to allow matrix polymerization. Subsequently, transfected HUVECs were seeded onto the polymerized Matrigel at a density of 1×10^5 cells per well. Endothelial tube formation was monitored and captured photographically at 3 and 6 h post-seeding.

Scratch wound migration assay

To assess cell migration, HUVECs were seeded into 6-cm dishes and cultured for 24 h to attain approximately 85% confluency, followed by transfection for HAND2 overexpression for an additional 24 h. A uniform mechanical scratch was introduced into the confluent cell monolayer using a sterile 200-μL pipette tip. Cellular debris and detached cells were removed by washing the monolayers twice with phosphate-buffered saline (PBS). To monitor migratory progress, phase-contrast micrographs of the wound area were acquired at 0, 12, and 24 h post-wounding.

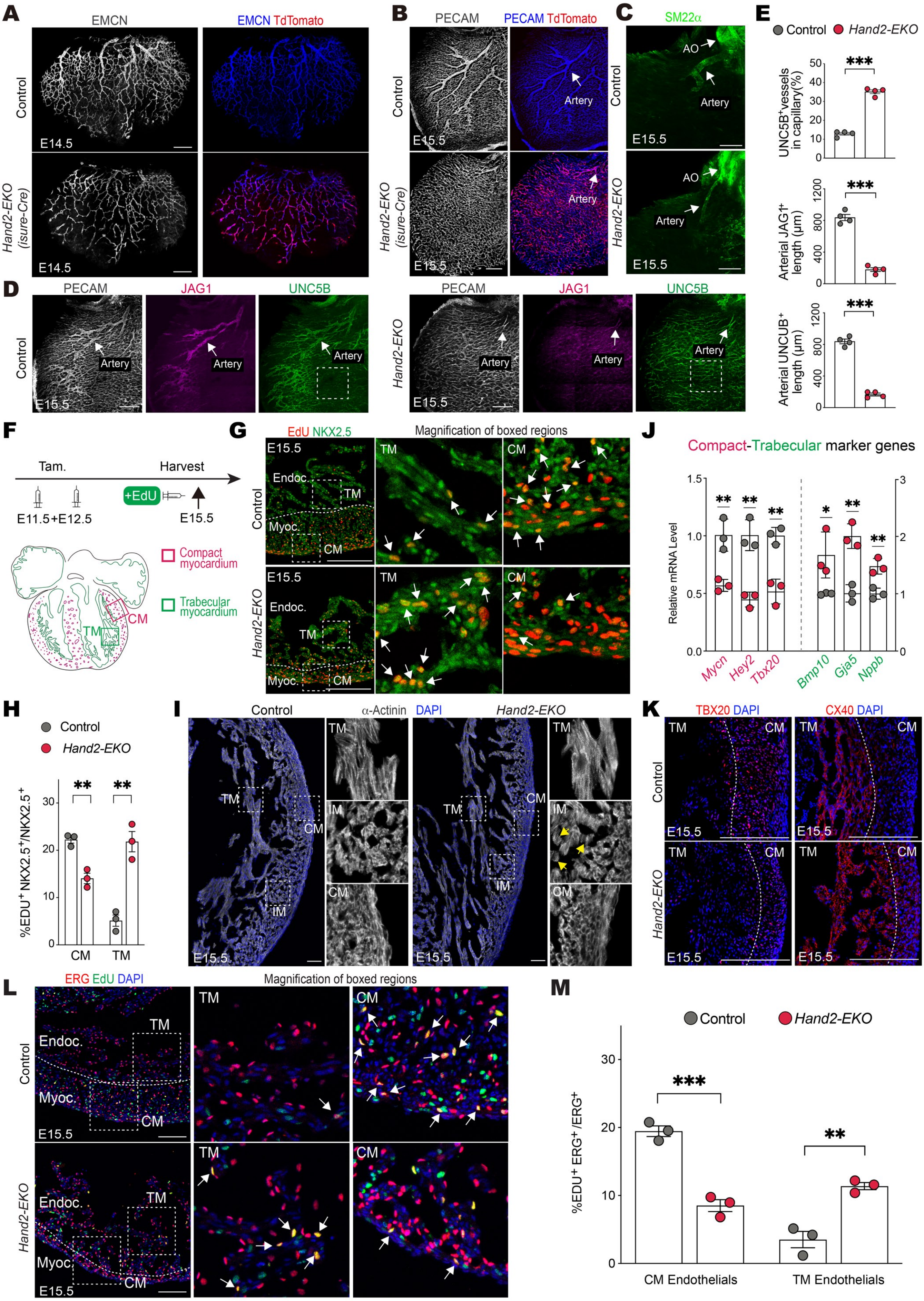
A
EMCN
EMCN TdTomato
Control
Hand2-EKO (isure-Cre)
E14.5
B
PECAM
PECAM TdTomato
Artery
E15.5
C
SM22α
AO
Artery
D
PECAM
JAG1
UNC5B
E
Control
Hand2-EKO
UNC5B+vessels in capillary(%)
Arterial JAG1+ length (μm)
Arterial UNCUB+ length (μm)
***
F
Tam.
Harvest
+EdU
E11.5+E12.5
E15.5
Compact myocardium
Trabecular myocardium
TM
CM
G
EdU NKX2.5
Magnification of boxed regions
Endoc.
Myoc.
J
Compact-Trabecular marker genes
Relative mRNA Level
Mycn
Hey2
Tbx20
Bmp10
Gja5
Nppb
H
%EDU+NKX2.5+/NKX2.5+
I
α-Actinin DAPI
IM
K
TBX20 DAPI
CX40 DAPI
L
ERG EdU DAPI
M
%EDU+ ERG+/ERG+
CM Endothelials
TM Endothelials

Supplemental Figure Legends

Supplemental Figure 1. Endothelial *Hand2* deletion impairs coronary angiogenesis and ventricular chamber development. (**A**) Wholemount staining showing defects of venous plexus morphogenesis in *Hand2-EKO; iSure-Cre* mice at E14.5. Immunostaining of TdTomato represents the cre recombination efficiency, n=3 hearts per group. (**B**) Wholemount staining showing absence of large coronary arteries in *Hand2-EKO; iSure-Cre* mice at E15.5, n=3 hearts per group. (**C**) Confocal images indicate normal connection between artery and aorta stem, but exhibits poorly artery development, n=3 hearts per group. AO, Aorta. (**D**-**E**) Representative images and quantitative analyses of arterial markers UNC5B and JAG1 showing lack of main artery formation, n=4 hearts per group. (**F**) Experimental strategy of *Hand2* ablation with Tamoxifen induction at E11.5-12.5 and samples harvested at E15.5. (**G**-**H**) Representative images (**G**) and quantitative analyses (**H**) of EdU positive (EdU^{+}) CMs and TMs from NKX2-5 and EdU immunofluorescence performed on E15.5 WT and *Hand2-EKO* hearts, n=3 hearts per group. (**I**) Immunostaining of a-Actinin reveals trabecular-like sarcomere morphology (yellow arrows) in the intermediate myocardium, n=3 hearts per group. (**J**) qPCR analysis of compact and trabecular myocardial marker genes of E15.5 WT and *Hand2-EKO* hearts, n=3 samples per group. (**K**) Confocal micrographs reveal downregulated CM marker TBX20 and upregulated TM marker CX40 expression in *Hand2-EKO* hearts. (**L**-**M**) Representative images (**L**) and quantitative analyses (**M**) of EdU positive (EdU^{+}) TMs and CMs from ERG and EdU immunofluorescence performed on E15.5 WT and *Hand2-EKO* hearts, n=3 hearts per group. Data represent mean ± SEM. Statistical significance was assessed using multiple unpaired two-tailed t tests with Benjamini–Krieger–Yekutieli FDR correction for (**H**) and (**M**), and unpaired two-tailed Student's t tests for (**E**) and (**J**).. $^{*}P < 0.05$, $^{**}P < 0.01$, $^{***}P < 0.001$. Scale bars (**A**, **B, C, D, G, I, K** and **L**), 200μm. CM compact myocardium, TM trabecular myocardium, IM intermediate myocardium, AO aorta.

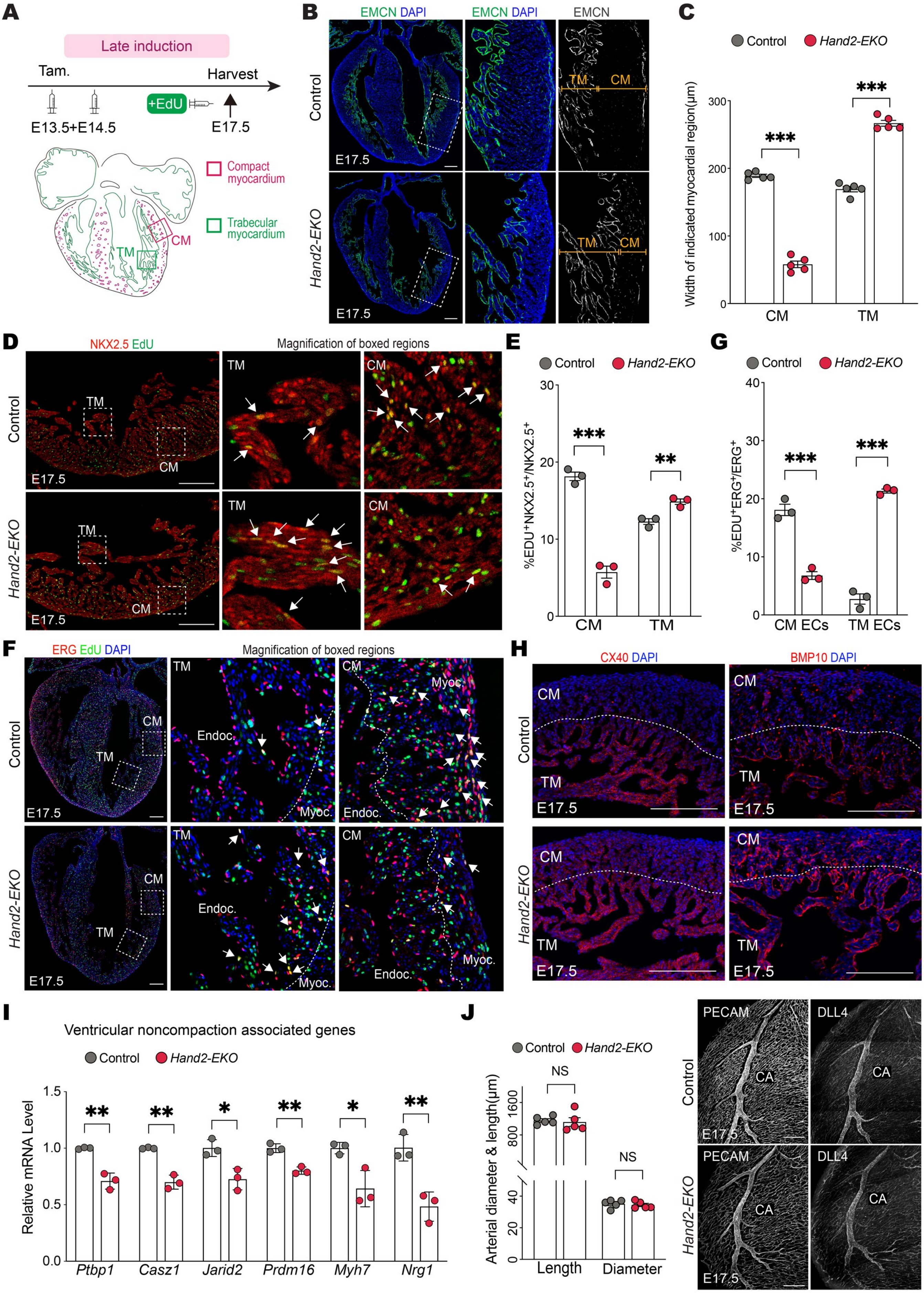
A
Late induction
Tam.
Harvest
+EdU
E13.5+E14.5
E17.5
Compact myocardium
Trabecular myocardium
TM
CM
B
EMCN DAPI
EMCN DAPI
EMCN
Control
Hand2-EKO
E17.5
TM
CM
C
Control
Hand2-EKO
Width of indicated myocardial region(μm)
***
***
CM
TM
D
NKX2.5 EdU
Magnification of boxed regions
Control
Hand2-EKO
E17.5
TM
CM
E
Control
Hand2-EKO
%EDU+NKX2.5+/NKX2.5+
***
**
CM
TM
G
Control
Hand2-EKO
%EDU+ERG+/ERG+
***
***
CM ECs
TM ECs
F
ERG EdU DAPI
Magnification of boxed regions
Control
Hand2-EKO
E17.5
TM
CM
Endoc.
Myoc.
H
CX40 DAPI
BMP10 DAPI
Control
Hand2-EKO
CM
TM
E17.5
I
Ventricular noncompaction associated genes
Control
Hand2-EKO
Relative mRNA Level
**
**
*
**
*
**
Ptbp1
Casz1
Jarid2
Prdm16
Myh7
Nrg1
J
Control
Hand2-EKO
Arterial diameter & length(μm)
NS
NS
Length
Diameter
PECAM
DLL4
CA
Control
Hand2-EKO
E17.5

Supplemental Figure 2. Late deletion of endothelial *Hand2* leads to ventricular noncompaction but independent of artery formation. (**A**) Experimental strategy of Hand2 ablation with Tamoxifen induction at E13.5-14.5 and samples harvested at E17.5. (**B**-**C**) Immunostaining (**B**) and quantitative analyses (**C**) of EMCN showing decreased myocardial thickness and increased trabecular layers in E17.5 *Hand2-EKO* hearts, n=5 hearts per group. (**D**-**E**) Representative images (**D**) and quantitative analyses (**E**) of EdU positive (EdU^{+}) TMs and CMs from Nkx2-5 and EdU immunofluorescence performed on E17.5 WT and *Hand2-EKO* hearts, n=3 hearts per group. (**F**-**G**) Representative images (**F**) and quantitative analyses (**G**) of EdU positive (EdU^{+}) TMs and CMs from ERG and EdU immunofluorescence performed on E17.5 WT and *Hand2-EKO* hearts, n=3 hearts per group. (**H**) Confocal images showing upregulation of well-known trabecular markers BMP10 and CX40 in *Hand2* mutants at E17.5, n=3 hearts per group. (**I**) qPCR analysis of ventricular noncompaction associated genes, n=3 samples per group. (**J**) Wholemount staining of DLL4 and quantitative analyses showing the normal artery morphology in E17.5 *Hand2-EKO* hearts, n=5 samples per group. Data represent mean ± SEM. Statistical significance was assessed using multiple unpaired two-tailed t tests with Benjamini–Krieger–Yekutieli FDR correction for (**C**), (**E**), (**G**) and (**J**), and an unpaired two-tailed Student's t test for (**I**). $^{*}P < 0.05$, $^{**}P < 0.01$, $^{***}P < 0.001$, NS not significant. Scale bars (**B**, **D**, **F**, **H** and **J**), 200μm. CM compact myocardium, TM trabecular myocardium, IM intermediate myocardium. CA coronary artery.

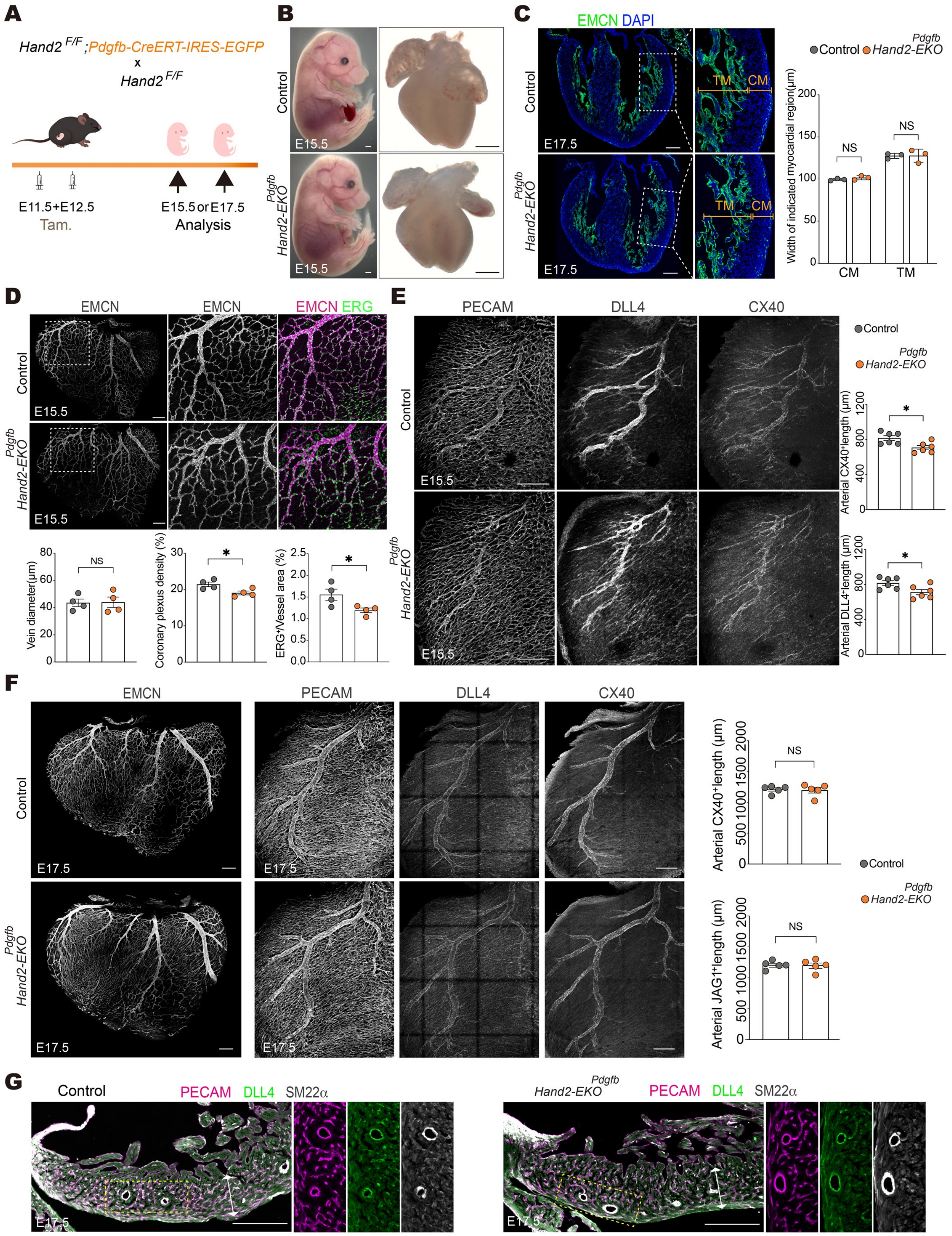

A
Hand2 F/F ;Pdgfb-CreERT-IRES-EGFP
x
Hand2 F/F
E11.5+E12.5
Tam.
E15.5 or E17.5
Analysis
B
Control
Pdgfb Hand2-EKO
E15.5
C
EMCN DAPI
Control
Pdgfb Hand2-EKO
E17.5
TM
CM
Control
Pdgfb Hand2-EKO
Width of indicated myocardial region(μm)
NS
D
EMCN
EMCN ERG
Control
Pdgfb Hand2-EKO
E15.5
Vein diameter(μm)
Coronary plexus density (%)
ERG+/Vessel area (%)
NS
*
E
PECAM
DLL4
CX40
Control
Pdgfb Hand2-EKO
E15.5
Arterial CX40+length (μm)
Arterial DLL4+length (μm)
*
F
EMCN
PECAM
DLL4
CX40
Control
Pdgfb Hand2-EKO
E17.5
Arterial CX40+length (μm)
Arterial JAG1+length (μm)
NS
G
Control
PECAM DLL4 SM22α
Pdgfb Hand2-EKO
E17.5

Supplemental Figure 3. Deficiency of *Hand2* in SV-derived ECs doesn't impair normal cardiovascular development. (**A**) Experimental strategy of *Hand2* ablation in coronary endothelium with *Pdgfb-creERT2* lineage. (**B**) Bright field images of E15.5 gross embryos and hearts. (**C**) Immunostaining of EMCN indicates the thickness of myocardial wall is normal in $\textit{Hand2-EKO}^{\textit{Pdgfb}}$ hearts, n=3 hearts per group. (**D**) Wholemount staining of EMCN and ERG in coronary vein showing relatively normal venous plexuses formation in E15.5 $\textit{Hand2-EKO}^{\textit{Pdgfb}}$ hearts with quantification analysis, n=4 hearts per group. (**E**) Wholemount staining of PECAM, DLL4 and CX40 in coronary artery showing relatively normal artery formation at E15.5 in $\textit{Hand2-EKO}^{\textit{Pdgfb}}$ hearts with quantification analysis, n=6 E15.5 hearts per group. (**F**) Wholemount staining for coronary artery markers (PECAM, DLL4, CX40) and coronary vein marker (EMCN) shows intact arteriovenous formation in E17.5 $\textit{Hand2-EKO}^{\textit{Pdgfb}}$ hearts with quantification analysis, n=5 E17.5 hearts per group. (**G**) Confocal images of E17.5 sectional analysis showing normal coronary arteries appear in *Hand2* mutants, n=3 hearts per group. Data represent mean ± SEM. Statistical significance was assessed using multiple unpaired two-tailed t test with Benjamini–Krieger–Yekutieli FDR correction for (**C**), and unpaired two-tailed Student's t tests for (**D**), (**E**), and (**F**). $^*P < 0.05$, NS not significant. Scale bars (**B**), 500μm. Scale bars (**C**, **D**, **E**, **F** and **G**), 200μm.

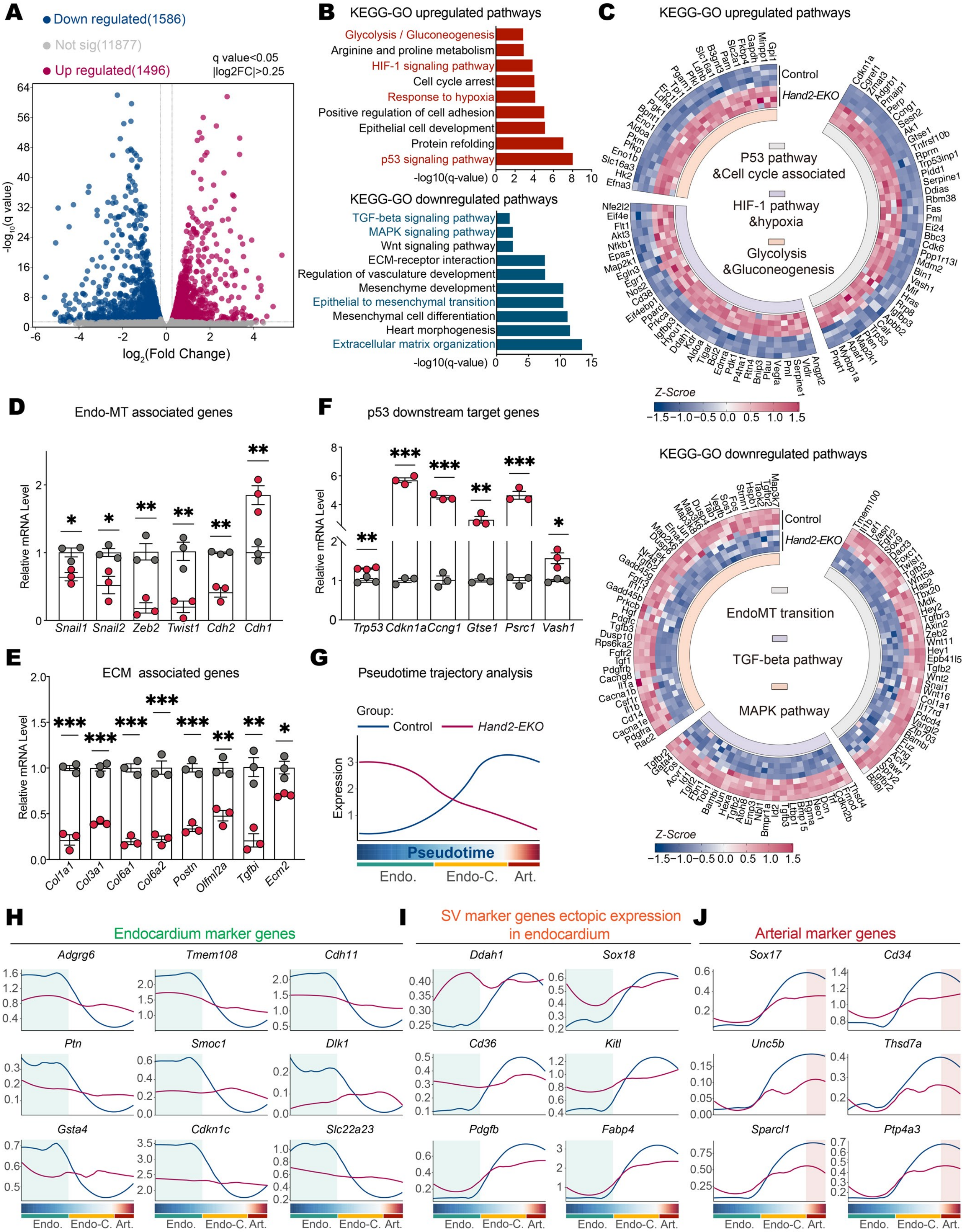

A
Down regulated(1586)
Not sig(11877)
Up regulated(1496)
q value<0.05
|log2FC|>0.25
-log10(q value)
log2(Fold Change)
B
KEGG-GO upregulated pathways
Glycolysis / Gluconeogenesis
Arginine and proline metabolism
HIF-1 signaling pathway
Cell cycle arrest
Response to hypoxia
Positive regulation of cell adhesion
Epithelial cell development
Protein refolding
p53 signaling pathway
-log10(q-value)
KEGG-GO downregulated pathways
TGF-beta signaling pathway
MAPK signaling pathway
Wnt signaling pathway
ECM-receptor interaction
Regulation of vasculature development
Mesenchyme development
Epithelial to mesenchymal transition
Mesenchymal cell differentiation
Heart morphogenesis
Extracellular matrix organization
-log10(q-value)
C
KEGG-GO upregulated pathways
Control
Hand2-EKO
P53 pathway &Cell cycle associated
HIF-1 pathway &hypoxia
Glycolysis &Gluconeogenesis
Z-Scroe
KEGG-GO downregulated pathways
Control
Hand2-EKO
EndoMT transition
TGF-beta pathway
MAPK pathway
Z-Scroe
D
Endo-MT associated genes
Relative mRNA Level
Snail1 Snail2 Zeb2 Twist1 Cdh2 Cdh1
E
ECM associated genes
Relative mRNA Level
Col1a1 Col3a1 Col6a1 Col6a2 Postn Olfml2a Tgfbi Ecm2
F
p53 downstream target genes
Relative mRNA Level
Trp53 Cdkn1a Ccng1 Gtse1 Psrc1 Vash1
G
Pseudotime trajectory analysis
Group:
Control
Hand2-EKO
Expression
Pseudotime
Endo.
Endo-C.
Art.
H
Endocardium marker genes
Adgrg6 Tmem108 Cdh11
Ptn Smoc1 Dlk1
Gsta4 Cdkn1c Slc22a23
I
SV marker genes ectopic expression in endocardium
Ddah1 Sox18
Cd36 Kitl
Pdgfb Fabp4
J
Arterial marker genes
Sox17 Cd34
Unc5b Thsd7a
Sparcl1 Ptp4a3
Endo. Endo-C. Art.

Supplemental Figure 4. RNA-seq analysis of E14.5 WT and *Hand2-EKO* ECs. (**A**) Volcano plot showing the DEGs between control and *Hand2* mutants. (**B**) GO terms and KEGG pathway analysis of DEGs, which were identified by the criteria of |log2FC| > 0.25 and q-value < 0.05, n=4 samples per group. (**C**) Circos heatmap showing dysregulated gene signatures based on GO and KEGG enrichment analyses in control and *Hand2-EKO* hearts., n=4 samples per group. (**D-F**) qPCR analysis of expression of Endo-MT related genes, ECM associated genes and p53 downstream target genes, n=3 samples per group. (**G**) Pseudotime trajectory analysis of the Endo - Endo-Cap - Art axis. (**H-J**) Dynamic expression of endocardium, coronary vessel, and arterial marker genes along the Endo- Endo-Cap -Art axis. Data represent mean ± SEM. Statistical significance was assessed using unpaired two-tailed Student's t-tests for (**D**), (**E**) and (**F**). *P < 0.05, **P < 0.01, ***P < 0.001. Endo, endocardium; Endo-cap, Endocardial-derived capillaries; Art, artery.

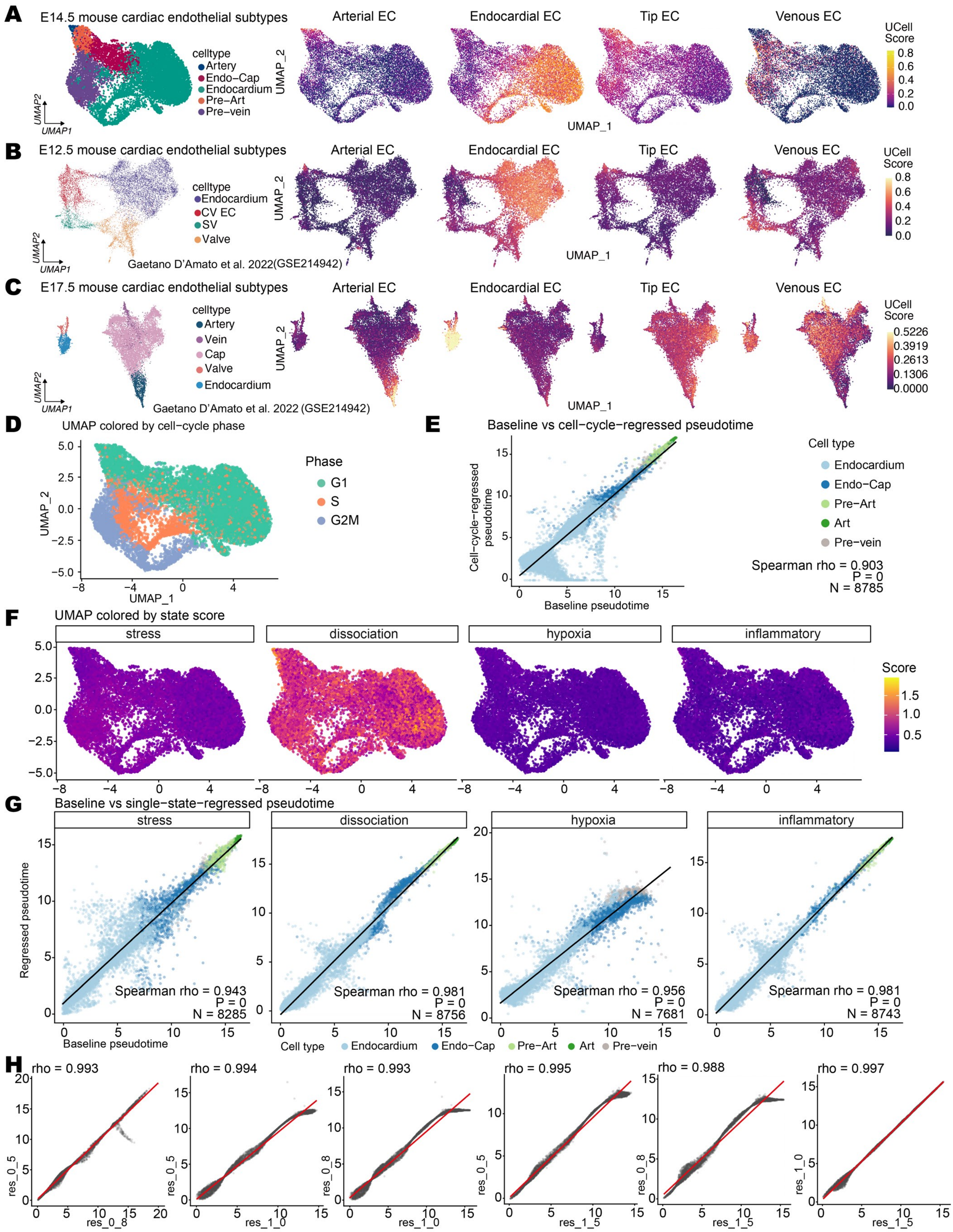

A
E14.5 mouse cardiac endothelial subtypes
celltype
Artery
Endo−Cap
Endocardium
Pre−Art
Pre−vein
UMAP2
UMAP1
Arterial EC
Endocardial EC
Tip EC
Venous EC
UMAP_2
UMAP_1
UCell Score
0.8
0.6
0.4
0.2
0.0
B
E12.5 mouse cardiac endothelial subtypes
celltype
Endocardium
CV EC
SV
Valve
Gaetano D'Amato et al. 2022(GSE214942)
Arterial EC
Endocardial EC
Tip EC
Venous EC
UCell Score
C
E17.5 mouse cardiac endothelial subtypes
celltype
Artery
Vein
Cap
Valve
Endocardium
Gaetano D'Amato et al. 2022 (GSE214942)
Arterial EC
Endocardial EC
Tip EC
Venous EC
UCell Score
0.5226
0.3919
0.2613
0.1306
0.0000
D
UMAP colored by cell-cycle phase
Phase
G1
S
G2M
UMAP_1
UMAP_2
E
Baseline vs cell−cycle−regressed pseudotime
Cell-cycle-regressed pseudotime
Baseline pseudotime
Cell type
Endocardium
Endo−Cap
Pre−Art
Art
Pre−vein
Spearman rho = 0.903
P = 0
N = 8785
F
UMAP colored by state score
stress
dissociation
hypoxia
inflammatory
Score
1.5
1.0
0.5
G
Baseline vs single−state−regressed pseudotime
stress
dissociation
hypoxia
inflammatory
Regressed pseudotime
Baseline pseudotime
Spearman rho = 0.943
P = 0
N = 8285
Spearman rho = 0.981
P = 0
N = 8756
Spearman rho = 0.956
P = 0
N = 7681
Spearman rho = 0.981
P = 0
N = 8743
Cell type Endocardium Endo−Cap Pre−Art Art Pre−vein
H
rho = 0.993
res_0_5
res_0_8
rho = 0.994
res_0_5
res_1_0
rho = 0.993
res_0_8
res_1_0
rho = 0.995
res_0_5
res_1_5
rho = 0.988
res_0_8
res_1_5
rho = 0.997
res_1_0
res_1_5

Supplemental Figure 5. Validation of scRNA-seq cluster annotation and robustness of endocardial–arterial trajectory inference. (**A**) Scoring analysis of well-known cardiac endothelial markers based on E14.5 scRNA-seq data in this study. (**B**-**C**) Publicly available E12.5 and E17.5 scRNA-seq datasets were retrieved from Gaetano D’Amato et al. 2022 (GSE214942). (**D**-**E**) Cell cycle phases span the entire trajectory (**D**), and regressing out cell cycle effects retains cell ordering (**E**). (**F**-**G**) Exclusion of stress, dissociation, hypoxia and inflammation confounding in the Slingshot pseudotime trajectory, shows weak correlation with pseudotime (**F**); regressing each score out independently preserves the arterial ordering (**G**). (**H**) Correlation analysis results under different resolutions. Pairwise correlation coefficients among all tested resolutions were higher than 0.99.

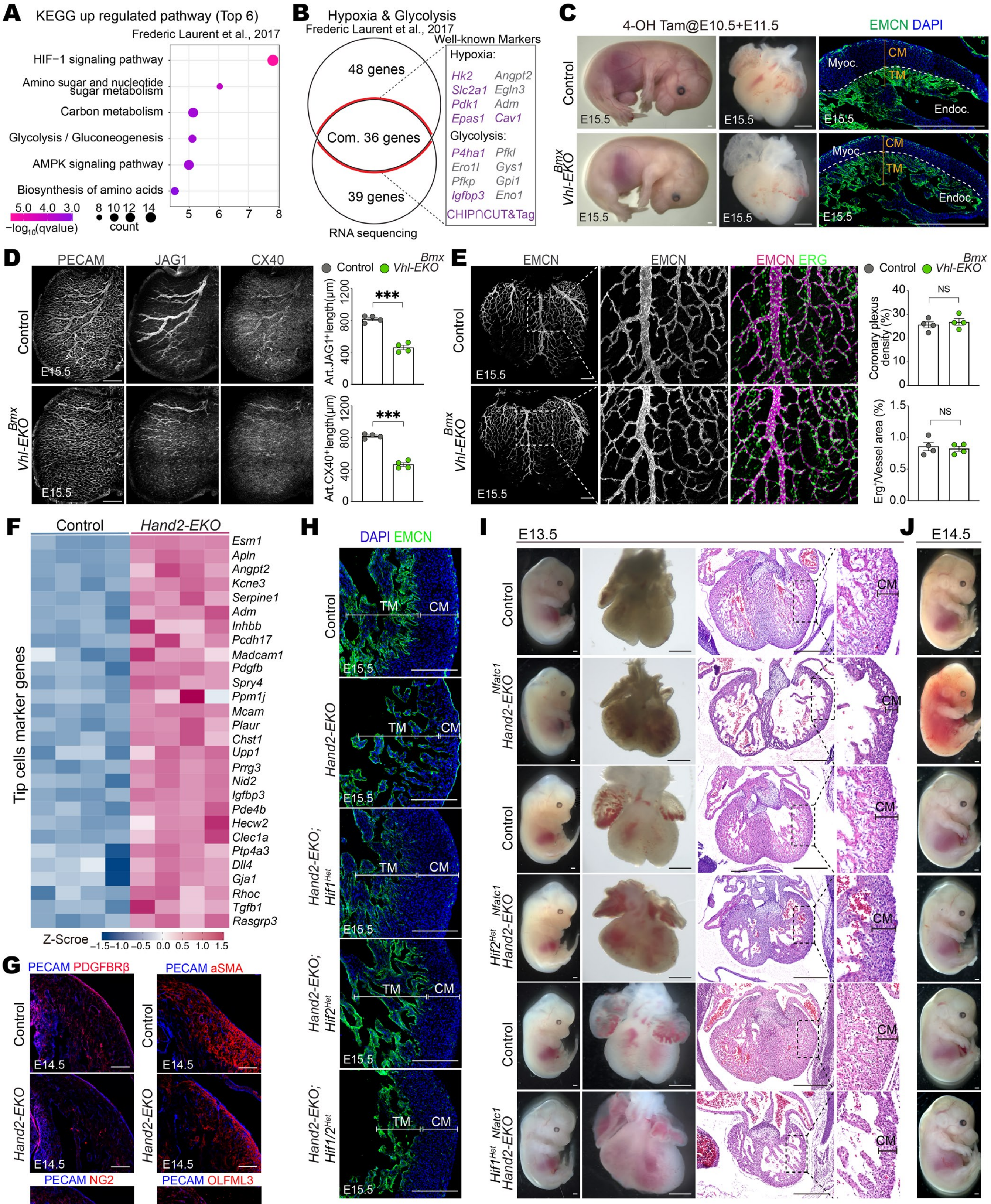

K

Survival table

Stage	Total No.	$Hand2\text{-}EKO^{Nfatc1}$ Survival Rate	Total No.	$Hif1^{Het}$ $Hand2\text{-}EKO^{Nfatc1}$ Survival Rate	Total No.	$Hif2^{Het}$ $Hand2\text{-}EKO^{Nfatc1}$ Survival Rate
E13.5	47	10/10(100%)	51	12/12(100%)	55	14/14(100%)
E14.5	55	1/12(11%)	60	8/13(62%)	47	6/10(60%)
E15.5	41	0/9(0%)	57	5/15(33%)	63	5/14(36%)

Supplemental Figure 6. Enhanced hypoxia pathway in *Hand2-EKO* hearts impairs cardiac development and mouse survival. (**A**) Bubble plot showing upregulated gene sets with KEGG analysis, DE genes were identified by the criteria of |log2FC| > 0.46 and q-value < 0.05. (**B**) Intersection circle plot showing hypoxia and glycolysis associated genes. Purple fonts indicate genes overlapped with CUT&Tag data. (**C**) Global and histological analysis showing defects of embryos and hearts in *Vhl-EKO*^{Bmx} mice. Immunostaining of EMCN indicates decreased myocardial wall thickness, n=3 hearts per group. (**D**) Confocal images and quantitative analysis showing coronary artery developmental and remodeling defects. Arteries are labeled with JAG1 and CX40, n=4 hearts per group. (**E**) Wholemount staining of EMCN and ERG in coronary vein showing normal venous plexuses formation with quantification analysis, n=4 hearts per group. (**F**) Heatmap analysis showing increased tip cell marker genes in *Hand2-EKO* mice. (**G**) Confocal images showing decreased expression of pericyte marker genes, including PDGFRb, aSMA, NG2 and OLFML3, n=3 hearts per group. (**H**) Immunostaining of sections from E15.5 controls, *Hand2-EKO*, and *Hand2/Hifs* mutants with EMCN indicates that myocardial development is relatively normal after repressing the HIF pathway, n=3 hearts per group. (**I**-**J**) Global and histological analyses of *Hand2* and *Hand2/Hifs* double mutant hearts at E13.5 and E14.5 with *Nfatc1-cre* lineage, n=3 hearts per group. (**K**) Survival table indicates extend lifespan in *Hand2-EKO*$^{Nfatc1\text{-}cre}$ mice after interrupting HIFs in endocardium. Data represent mean ± SEM. Statistical significance was assessed using unpaired two-tailed Student's t-tests for (**D**) and (**E**). ***P < 0.001, NS not significant. Scale bars (**C**, **I** and **l**), 500μm. Scale bars (**D**, **E** and **G**), 200μm.

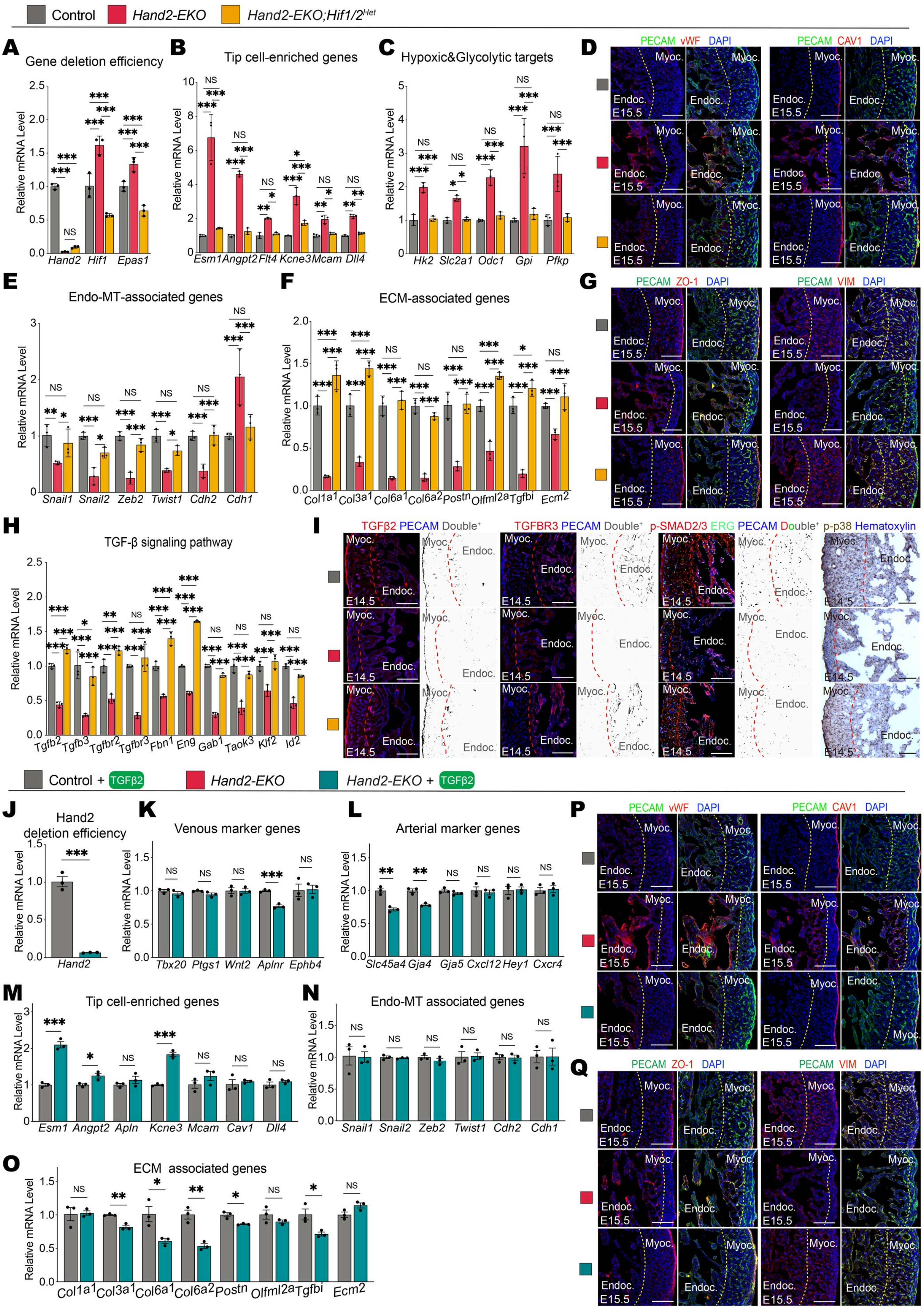

Control
Hand2-EKO
Hand2-EKO;Hif1/2Het
A
Gene deletion efficiency
B
Tip cell-enriched genes
C
Hypoxic&Glycolytic targets
D
E
Endo-MT-associated genes
F
ECM-associated genes
G
H
TGF-β signaling pathway
I
Control + TGFβ2
Hand2-EKO
Hand2-EKO + TGFβ2
J
Hand2 deletion efficiency
K
Venous marker genes
L
Arterial marker genes
M
Tip cell-enriched genes
N
Endo-MT associated genes
O
ECM associated genes
P
Q
Relative mRNA Level
Hand2 Hif1 Epas1
Esm1 Angpt2 Flt4 Kcne3 Mcam Dll4
Hk2 Slc2a1 Odc1 Gpi Pfkp
PECAM vWF DAPI
PECAM CAV1 DAPI
PECAM ZO-1 DAPI
PECAM VIM DAPI
Myoc.
Endoc.
E15.5
E14.5
Snail1 Snail2 Zeb2 Twist1 Cdh2 Cdh1
Col1a1 Col3a1 Col6a1 Col6a2 Postn Olfml2a Tgfbi Ecm2
Tgfb2 Tgfb3 Tgfbr2 Tgfbr3 Fbn1 Eng Gab1 Taok3 Klf2 Id2
TGFβ2 PECAM Double+
TGFBR3 PECAM Double+
p-SMAD2/3 ERG PECAM Double+
p-p38 Hematoxylin
Tbx20 Ptgs1 Wnt2 Aplnr Ephb4
Slc45a4 Gja4 Gja5 Cxcl12 Hey1 Cxcr4
Esm1 Angpt2 Apln Kcne3 Mcam Cav1 Dll4
NS

Supplemental Figure 7. Suppression of HIF activity or reinforcement of TGF-signalling partially restore *Hand2-EKO* defects at the molecular levels. (**A**-**B**) qPCR analysis of *Hand2, Hif1a, Hif2a* and metabolism-associated genes in E14.5 control, *Hand2-EKO*, and *Hand2/Hifs* mutant cardiac ECs, n=3 per group. (**C**-**D**) qPCR analysis of tip cell-enriched genes in cardiac ECs (**C**); n = 3 per group. Cardiac sections immunostained for vWF and CAV1 (**D**); n = 3 per group. (**E**-**G**) qPCR analysis of Endo-MT- and ECM-associated genes in cardiac ECs (**E**, **F**), n = 3 per group. Cardiac sections immunostained for ZO-1 and VIM (**G**), n = 3 per group. (**H**-**I**) qPCR analysis of TGF-β signaling pathway associated genes in cardiac ECs (**H**), n = 3 per group. Cardiac sections immunostained for TGFβ2, TGFBR3, P-SMAD2/3 and P-P38 (**I**), n = 3 per group. (**J**) qPCR analysis showing *Hand2* deletion efficiency after TGFβ2 treatment in E14.5 *Hand2-EKO* and control cardiac ECs, n=3 per group. (**K**-**L**) qPCR analysis shows that dysregulated venous and arterial gene expression in E14.5 *Hand2-EKO* cardiac ECs was partially rescued following 2.25μg TGFβ2 treatment, n=3 per group. (**M**-**Q**) qPCR analysis of tip cell-enriched, Endo-MT- and ECM-associated genes in cardiac ECs, together with immunofluorescence staining of cardiac sections for vWF/CAV1 and ZO-1/VIM; n = 3 per group for all assays. Data represent mean ± SEM. Statistical significance was assessed using an unpaired two-tailed Student's t-test (**J**, **K**, **L**, **M**, **O** and **P**) or two-way ANOVA (**A**, **B**, **C**, **E**, **F** and **H**) followed by Tukey's multiple-comparison test. *$P < 0.05$, **$P < 0.01$, ***$P < 0.001$, NS not significant. Scale bars (**D**, **G**, **I**, **P** and **Q**), 100μm.

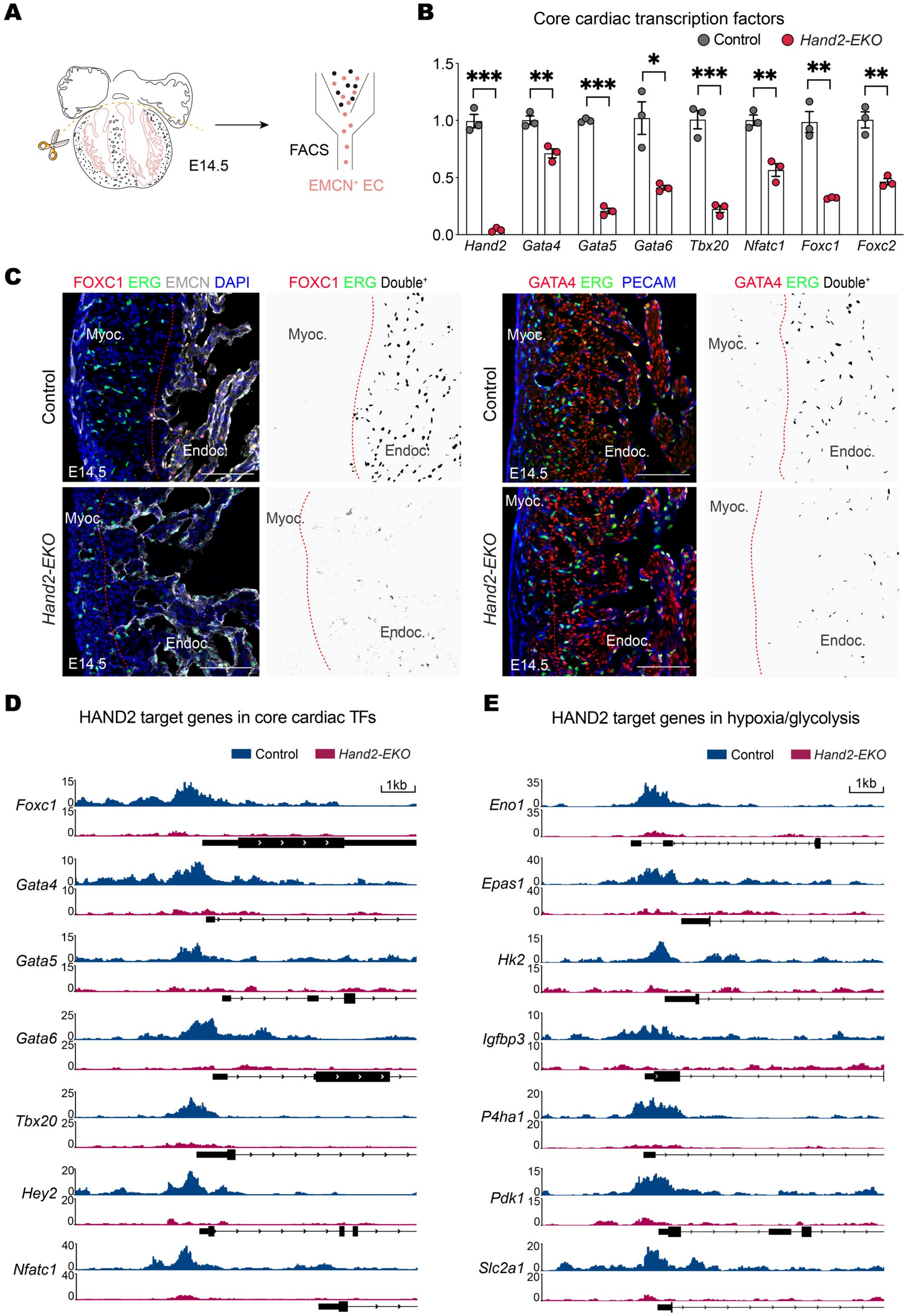
A
E14.5
FACS
EMCN+ EC
B
Core cardiac transcription factors
Control
Hand2-EKO
Hand2
Gata4
Gata5
Gata6
Tbx20
Nfatc1
Foxc1
Foxc2
C
FOXC1 ERG EMCN DAPI
FOXC1 ERG Double+
GATA4 ERG PECAM
GATA4 ERG Double+
Control
Hand2-EKO
Myoc.
Endoc.
E14.5
D
HAND2 target genes in core cardiac TFs
Control
Hand2-EKO
1kb
Foxc1
Gata4
Gata5
Gata6
Tbx20
Hey2
Nfatc1
E
HAND2 target genes in hypoxia/glycolysis
Eno1
Epas1
Hk2
Igfbp3
P4ha1
Pdk1
Slc2a1

Supplemental Figure 8. HAND2 physically interacts with promoters of core cardiac TFs and hypoxia-associated genes. (**A**) Schematic illustration of strategies of EMCN$^+$ ECs sorting for qPCR analysis. (**B**) qPCR analysis of core cardiac transcription factors in EMCN$^+$ sorted ECs, n=3 samples per group. (**C**) Confocal images showing decreased expression of key TFs, including FOXC1 and GATA4, n=3 hearts per group. (**D**) CUT&Tag tracks of core cardiac transcription factors loci (*Foxc1*, *Gata4*, *Gata6*, *Tbx20*, *Hey2* and *Nfatc1*) in control and *Hand2-KO* groups. (**E**) CUT&Tag tracks of hypoxia& glycolysis genes loci (*Eno1*, *Epas1*, *Hk2*, *Igfbp3*, *P4ha1*, *Pdk1* and *Slc2a1*) in control and *Hand2-KO* groups. Data represent mean ± SEM. Statistical significance was assessed using an unpaired two-tailed Student's t-test for (**B**). $^*P < 0.05$, $^{**}P < 0.01$, $^{***}P < 0.001$. Scale bars (**C**), 100μm.

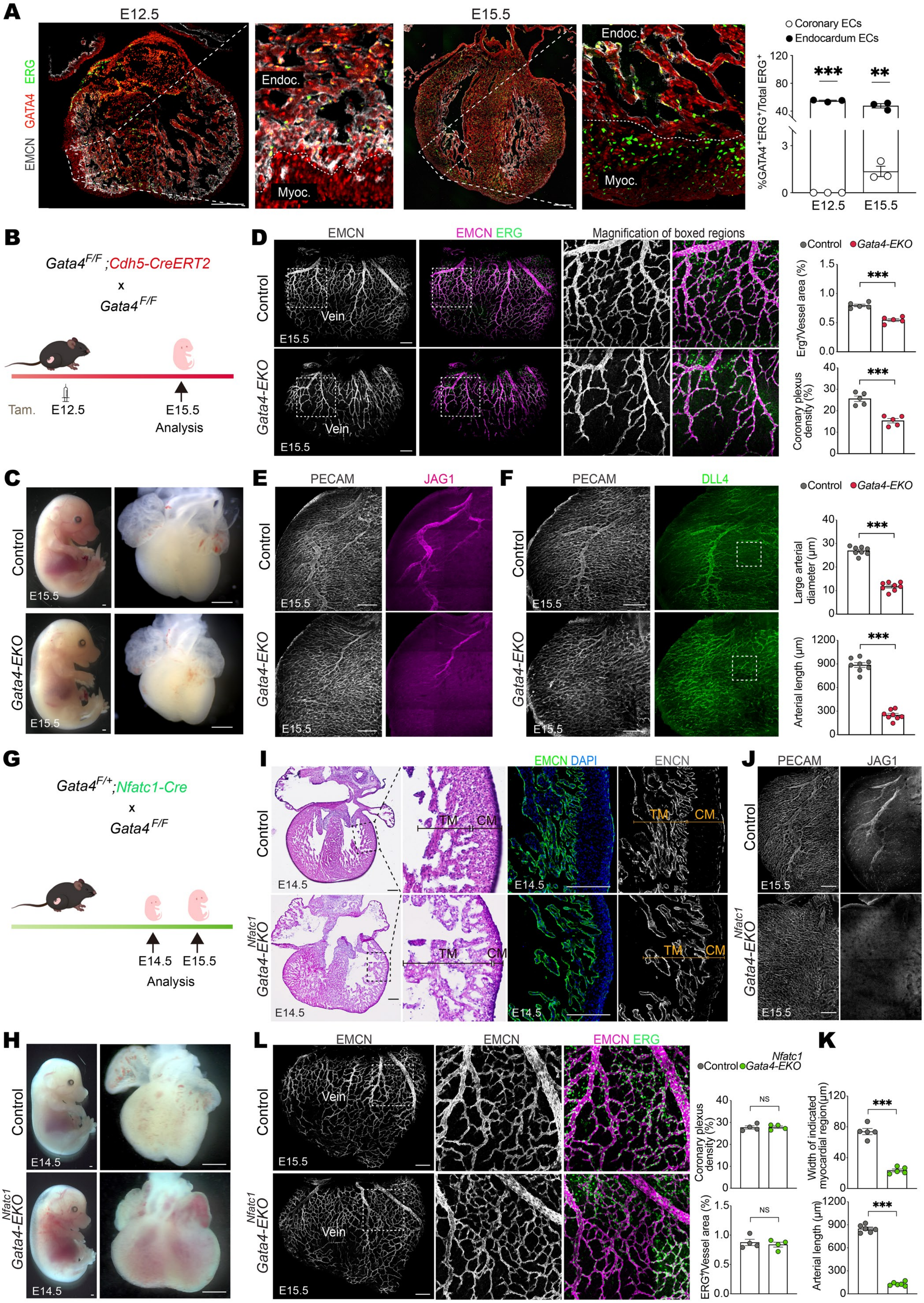

A
E12.5
E15.5
EMCN GATA4 ERG
Endoc.
Myoc.
Coronary ECs
Endocardum ECs
%GATA4+ERG+/Total ERG+
E12.5
E15.5
B
Gata4F/F;Cdh5-CreERT2
x
Gata4F/F
Tam.
E12.5
E15.5
Analysis
C
Control
Gata4-EKO
E15.5
D
EMCN
EMCN ERG
Magnification of boxed regions
Vein
Control
Gata4-EKO
Erg+/Vessel area (%)
Coronary plexus density (%)
E
PECAM
JAG1
F
DLL4
Large arterial diameter (μm)
Arterial length (μm)
G
Gata4F/+;Nfatc1-Cre
x
Gata4F/F
E14.5
E15.5
Analysis
H
Nfatc1 Gata4-EKO
I
EMCN DAPI
ENCN
TM
CM
E14.5
J
PECAM
JAG1
L
EMCN
EMCN ERG
NS
ERG+/Vessel area (%)
K
Width of indicated myocardial region(μm)
Arterial length (μm)

Supplemental Figure 9. Expression pattern of endothelial *Gata4* and cardiovascular defects in *Gata4* mutants. (**A**) Confocal images showing endothelial *Gata4* were specifically restricted in endocardium in E12.5 and E15.5 hearts, n=3 hearts per group. (**B**) Experimental strategy for *Gata4* deletion in cardiac endothelium. (**C**) Bright field images of E15.5 gross embryos and hearts showing defective embryogenesis. (**D**) Wholemount staining of EMCN and ERG in coronary vein showing decreased density of coronary veins and ERG numbers in E15.5 *Gata4-EKO* hearts with quantification analysis, n=5 hearts per group. (**E**-**F**) Wholemount staining of JAG1 and DLL4 in coronary artery showing coronary artery defects in E15.5 *Gata4-EKO* hearts, n=8 hearts per group. (**G**) Experimental strategy of *Gata4* deletion in endocardium with *Nfatc1-cre* lineage. (**H**) Bright field images of E14.5 gross embryos and hearts showing defective embryogenesis and dilated hearts. (**I**) Histological and Immunostaining analyses showing thinning of myocardial wall in *Gata4-EKO* hearts. (**J**) Wholemount staining of JAG1 showing defects of coronary artery extension and branching. (**K**) Quantification analysis of (**I**), n=5 hearts per group. and (**J**), n=6 hearts per group. (**L**) Wholemount staining of EMCN and ERG in coronary vein showing relative normal venous plexuses formation in E15.5 *Gata4-EKO*^{Nfatc1} hearts with quantification analysis, n=4 hearts per group. Data represent mean ± SEM. Statistical significance was assessed using two-tailed paired t-test with Benjamini–Krieger–Yekutieli false discovery rate (FDR) correction for (**A**), and unpaired two-tailed Student's t tests for (**D**), (**F**), (**K**) and (**L**). $^{**}P < 0.01$, $^{***}P < 0.001$, NS not significant. Scale bars (**A**, **D**, **E**, **F**, **I**, **J** and **L**), 200µm. Scale bars (**C** and **H**), 500µm.

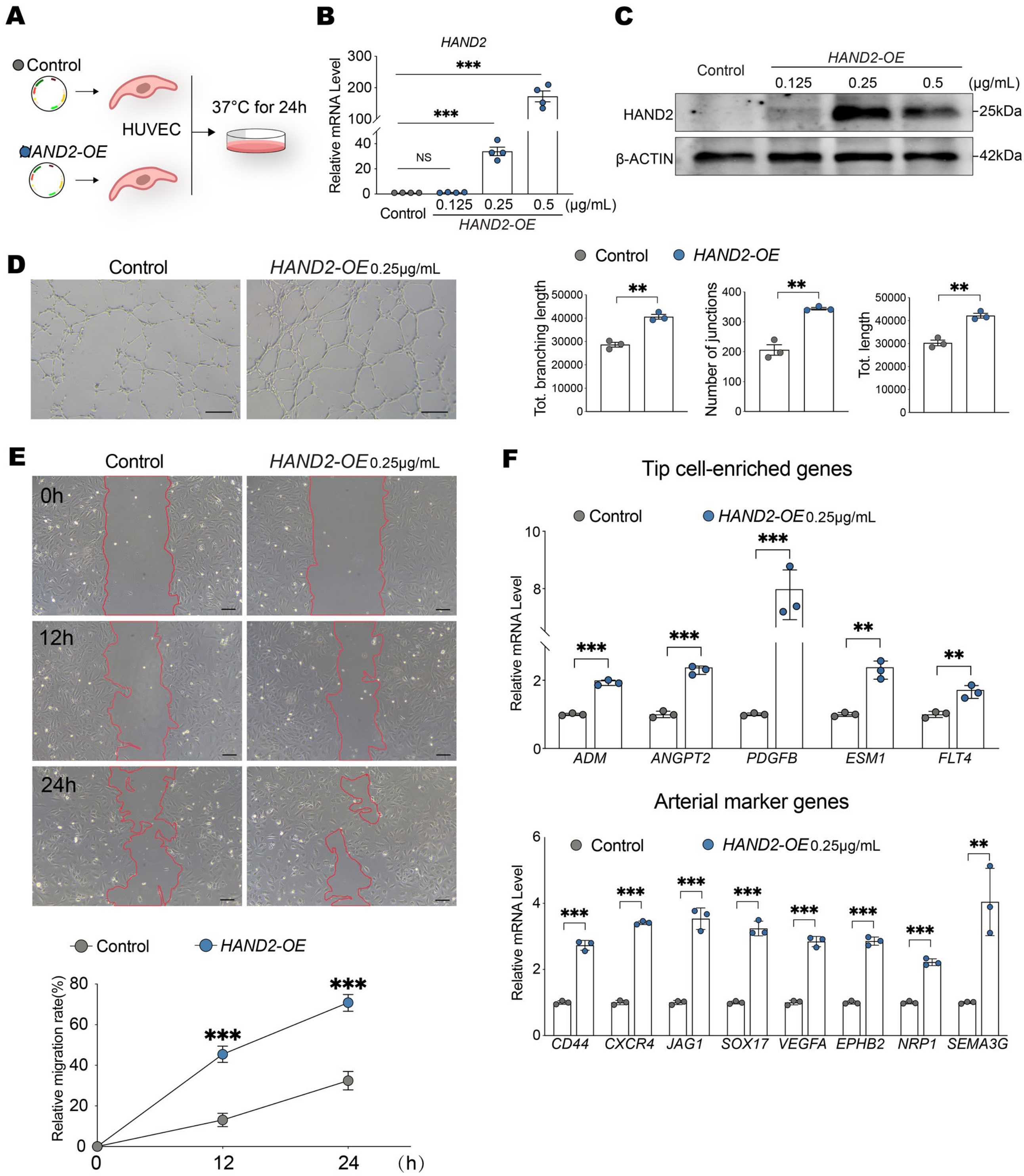
A
Control
HUVEC
37°C for 24h
HAND2-OE
B
HAND2
Relative mRNA Level
NS
***
Control
0.125
0.25
0.5
(μg/mL)
HAND2-OE
C
Control
HAND2-OE
0.125
0.25
0.5
(μg/mL)
HAND2
β-ACTIN
25kDa
42kDa
D
Control
HAND2-OE 0.25μg/mL
Tot. branching length
Number of junctions
Tot. length
**
E
Control
HAND2-OE 0.25μg/mL
0h
12h
24h
Control
HAND2-OE
Relative migration rate(%)
***
(h)
F
Tip cell-enriched genes
Control
HAND2-OE 0.25μg/mL
Relative mRNA Level
ADM
ANGPT2
PDGFB
ESM1
FLT4
Arterial marker genes
CD44
CXCR4
JAG1
SOX17
VEGFA
EPHB2
NRP1
SEMA3G

Supplemental Figure 10. Overexpression of *HAND2* in HUVECs promotes EC angiogenesis and arterialization. (**A**) Experimental strategy for *HAND2* overexpression in HUVECs. (**B**-**C**) qPCR and western blotting validation of *HAND2* mRNA and protein expression in HUVECs. (**D**-**E**) Tube formation assay in HUVECs and quantification analysis of branching length, length, and junctions after *HAND2* overexpression, n=3 samples per group. Scratch assays in HUVECs and relative migration rate quantification after *HAND2* overexpression, n=3 samples per group. (**F**) qPCR analysis of tip cell-enriched genes and arterial marker genes, n=3 samples per group. Data represent mean ± SEM. Statistical significance was assessed using one-way ANOVA for (**B**), two-way ANOVA for (**E**), followed by Tukey's multiple-comparison test, and unpaired two-tailed Student's t tests for (**F**). **P < 0.01, ***P < 0.001, NS not significant. Scale bars (**D**, **E**), 100μm

Supplemental Table1: Genotyping primers

Genotype	Primer	
Hand2 Floxed	For: CTGCAATAGCAGGAAGGGAA	WT band:320bp<br>Mut band:480 bp
	Rev: CGAGAGGGAGAGAAATGGAAAG	
Vhl Floxed	F1: CTGGTACCCACGAAACTGTC	WT band:300bp<br>Mut band:400 bp
	F2: CTAGGCACCGAGCTTAGAGGTTTGCG	
	R: CTGACTTCCACTGATGCTTGTCACAG	
Hif1 Floxed	For: CGTGTGAGAAAACTTCTGGATG	WT band:600 bp<br>Mut band:630 bp
	Rev: AAAAGTATTGTGTTGGGGCAGT	
Hif2 Floxed	For: GAGAGCAGCTTCTCCTGGAA	WT band:180 bp<br>Mut band:220 bp
	Rev: TGTAGGCAAGGAAACCAAGG	
Gata4 Floxed	For: CTGTTTTCATGAGCTCTAGGTTCC	WT band:162 bp<br>Mut band:227 bp
	Rev: TAGAATTCTCAACCATTGGGTGGC	
PAC-Cdh5-CreERT2	For: GACTAGCTTACCATTCTGCTGGTGT	Mut band: 300bp
	Rev: GGACAGAAGCATTTTCCAGGTATG	
PAC Pdgfb-iCre-ERT2-IresEGFP	PdgiCre-For: GCCGCCGGGATCACTCTCG	Mut band: 450bp
	PdgiCre-rev: CCAGCCGCCGTCGCAACTC	
Bmx-CreERT2	For: AAATACCTTCAGTTTTCATCT	Mut band: 750bp
	Rev: TTGCGAACCTCATCACTCGTT	
Nfatc1-Cre	5arm_Sense: AATAAGCCTGCCGTGGTCACTGG	WT band: 300bp<br>Mut band: 1000bp
	3arm_Antis: AACCCTGGACGCCTGGGACAC	
	CRE_Sense:GAAGCAACTCATCGATTGATTTACG	
iSure Cre	Sv40 pA F: CCCCCTGAACCTGAAACATA	WT band:104bp<br>Mut band: 260bp
	AVT Tomato R: CCTTGCTCACCATGGTCTTG	
	Chr 17 R: GTGTCTGTACCAGGTTGGTTTG	
	Chr 17 F: GCTCTGCATGTTGCAAGAAA	

Supplemental Table 2: Tamoxifen inductions and dissection stages

Genotype	Tamoxifen injection	Dosing	Analysis
Hand2$^{F/F}$*; Cdh5-CreERT2*	E11.5 and E12.5	4mg Tamoxifen	E15.5
Hand2$^{F/F}$*; Cdh5-CreERT2*	E13.5 and E14.5	4mg Tamoxifen	E17.5
Hand2$^{F/F}$*; HIF1*$^{F/+}$*; Cdh5-CreERT2*	E11.5 and E12.5	4mg Tamoxifen	E15.5
Hand2$^{F/F}$*; HIF2*$^{F/+}$*; Cdh5-CreERT2*	E11.5 and E12.5	4mg Tamoxifen	E15.5
Hand2$^{F/F}$*; HIF1*$^{F/+}$*; HIF2*$^{F/+}$*; Cdh5-CreERT2*	E11.5 and E12.5	4mg Tamoxifen	E15.5
Hand2$^{F/F}$*; Pdgfb-CreERT2*	E11.5 and E12.5	4mg Tamoxifen	E15.5 or E17.5
Hand2$^{F/F}$*; Bmx-CreERT2*	E10.5 and E11.5	3mg 4-OH Tamoxifen	E15.5
Hand2$^{F/F}$*; Bmx-CreERT2*	E11.5	1.5mg 4-OH Tamoxifen	E12.0(Explant)
Hand2$^{F/F}$*; Bmx-CreERT2*	E10.5	1.5mg 4-OH Tamoxifen	E15.5(Lineage tracing)
Bmx-CreERT2	E11.5	1.5mg 4-OH Tamoxifen	E12.0(Explant)
Bmx-CreERT2	E10.5	1.5mg 4-OH Tamoxifen	E15.5(Lineage tracing)
Hand2$^{F/F}$*; Nfatc1-Cre*			E13.5 to E15.5
Hand2$^{F/F}$*; HIF1*$^{F/+}$*; Nfatc1-Cre*			E13.5 to E15.5
Hand2$^{F/F}$*; HIF2*$^{F/+}$*; Nfatc1-Cre*			E13.5 to E15.5
Vhl$^{F/F}$*; Bmx-CreERT2*	E10.5 and E11.5	3mg 4-OH Tamoxifen	E15.5
Gata4$^{F/F}$*; Nfatc1-Cre*			E14.5 or E15.5
Gata4$^{F/F}$*; Cdh5-CreERT2*	E12.5	2mg Tamoxifen	E15.5

Supplemental Table 3: List of antibodies

Antibodies	SOURCE	IDENTIFIER
Anti-Pecam1	BD Biosciences	Cat# 551262
Anti-HAND2	Abcam	Cat# ab200040
Anti-CD31 APC	BD biosciences	Cat# ab551262
Anti-endomucin eFluor 660	Thermo Fisher Scientific	Cat# 50-5851-82
Anti-ERG-AF-647	Abcam	Cat# ab196149
Anti-Nkx2.5	santa cruz	Cat# sc-14033
Anti-endomucin	santa cruz	Cat# sc-53941
Anti-DLL4	R&D Systems	Cat# AF1389
Anti-CX40	Alpha Diagnostic	Cat# CX40-A
Anti-Plvap	BD biosciences	Cat# 550563
Anti-FOXC1	Cell Signaling Technology	Cat# 8758s
Anti-GATA4	santa cruz	Cat# sc-9053
Anti-FABP4	abclonal	Cat# A2579（20ul）
Anti-BMP10	R&D Systems	Cat# MAB6038
Anti-VE-cadherin	R&D Systems	Cat# 555289
Anti-ZO-1	Thermo Scientific	Cat# 33-9100
Anti-vimentin	affinity	Cat# bf8006
Anti-N-cadherin	abcam	Cat# ab98952
Anti-SLUG	Howard hughes medical institute	Cat# '62.1E6
Anti-p-SNAIL	bioworld	Cat# BS4606
Anti-HIF-1α	Cell Signaling Technology	Cat# 36169S
Anti-HIF-2α	Novus Biologicals	Cat# NB100-122
Anti-GLUT1	HUABIO	Cat# ET1601-10
Anti-ENO1	HUABIO	Cat# ET1705-56
Anti-Hexokinase II	HUABIO	Cat# HA500186
Anti-LDHA	HUABIO	Cat# ER00702
Anti-PDK1	HUABIO	Cat# ET1704-66
Anti-Triosephosphate isomerase	HUABIO	Cat# HA500283
Anti-Angiopoietin 2	aifang	Cat# AF301451
Anti-VWF	Millipore	Cat# AB7356
Anti-Caveolin 1	BD Biosciences	Cat# 610407
Anti-p-SMAD2/3	Cell Signaling Technology	Cat# 8828S
Anti-SMAD2/3	Aifang	Cat# AF03499
Anti-β-ACTIN	Proteintech	Cat# 66009-1-Ig
Anti-phospho-p38(T180/Y182) MAPK	Cell Signaling Technology	Cat# 9211L
Anti-p38 MAPK	Cell Signaling Technology	Cat# 9212
Anti-Phospho-p44/42MAPK(Erk1/2) (Thr202/Tyr204)	Cell Signaling Technology	Cat# 9101s
Anti-ERK	Cell Signaling Technology	Cat# 9102s

Anti-p-JNK	Cell Signaling Technology	Cat# 9251S
Anti-JNK	Cell Signaling Technology	Cat# 9252
Anti-PDGFRB	Invitrogen	Cat# 14-1402-82
Anti-SM22 alpha antibody	abcam	Cat# ab14106
Anti-JAG1	R&D Systems	Cat# AF599
Anti-UNC5B	Cell Signaling Technology	Cat# 13851S
Anti-E-cadherin	bioworld	Cat# BS1098
Anti-N-cadherin	santa cruz	Cat# SC-7939
Anti-NG2	Millipore	Cat# AB5320
Anti-OLFML3	Abiocode	Cat# R3884-2
Anti-TAB1	HUABIO	Cat# HA500400
Anti-aSMA	SIGMA	Cat# C6198
Anti-TGFβ2	santa cruz	Cat# sc-374658
Anti-TGFBR3	R&D Systems	Cat# AF242-PB

Supplemental Table 4: qPCR primers

MOUSE	Primer	*MOUSE*	Primer	*MOUSE*	Primer
Slc45a4	TGCCGACTCGGAATCTATGC	*Pkm*	CAAGGGGACTACCCTCTGG	*Fosl2*	TCTTCCTCAGCAGGGATGGA
	AACCAGTGCTGTCTCCATCG		ACACGAAGGTCGACATCCTC		CTTCCCCGTAGAAACCACCC
Gja4	TCCCACATCCGATACTGGGT	*Ldha*	TGTCTCCAGCAAAGACTACTGT	*Ddit3*	GTCACACGCACATCCCAAAG
	CCCGCCGAGACAGGTAGAT		GACTGTACTTGACAATGTTGGGA		GGGCACTGACCACTCTGTTT
Gja5	TTGGCAAGATGGGTGACTGG	*Gpi*	TTTCCTTCCTCTTCCGAGCAC	*Klf2*	TAAAGGCGCATCTGCGTACA
	GGACCAGCATGCGGAAAATG		AGGTTCAAGCTGAAGTTGTTGA		GTGGCACTGAAAGGGTCTGT
Cxcl12	TGCATCAGTGACGGTAAACCA	*Pgk1*	GTCGTGATGAGGGTGGACTT	*Klf4*	CTAACCGTTGGCGTGAGGAA
	TTCTTCAGCCGTGCAACAATC		AAGGACAACGGACTTGGCTC		CGGGTTGTTACTGCTGCAAG
Hey1	CATGAAGAGAGCTCACCCAGA	*Pfkp*	TATGGGAGTGGAGGCTGTCA	*Klf10*	CCCCTCCTCTTCCTAAGGCT
	CGCCGAACTCAAGTTTCC		CGGCTACATTGCAGTTGCTC		TTTCCGTGGGTTCTTCTCCG
Gata4	GTGGCCCTGGCGCCTTCATG	*Pfkl*	TGCATGATGGCTTTGAGGGT	*Gab1*	TTCCCATGAACCCCAACTCG
	TCCCAGGCCCTGCACCCGAC		GTGGATGTTGTAGGTGCGGA		ATGAGCAGGAGGAGGGACTT
Gata5	ATCCCGAAGATCCGGCCTAT	*Fbn1*	TGAACTGGACCGAAGTGGTG	*Rps6ka2*	CTCCCAAGGGGTTGTCCATC
	AGGGTTCTTTGGCTTCCGTT		AAATCCGGAGGACAATCGCA		TTCTCCGCTCGAAGCTGTTT
Gata6	TCGACTTGGGAGGACCTGTT	*Ptbp1*	TCCCAGACATAGCAGTCGGTACA	*Rps6ka5*	GTGCTTCACCCTCCACTACG
	TGGTACATTTCCTCCGGCTG		TGACATGGATGACTCTGGAAGG		CCTGCCCTGACAGCATTGTA
Tbx20	TTACCGGGTGTTTGCTTCCC	*Casz1*	GCTCATCAACGATGGCTTCCAG	*Taok3*	AGGAAGCAACCTGAACCGAG
	AGTGACAGGGCACTCACATG		GTGGTAGCTCTTGTGCTTCTCG		GTGCCTTCATCCTCCTGCAT
Hand2	GAGAACCCCTACTTCCACGG	*Jarid2*	GCCTGCATAAAGAAGCACCTCAG	*Rap2a*	GACTCCTCGCCTTCGGTG
	GACAGGGCCATACTGTAGTCG		GAGGTCAGTCACTTGCTGCATG		TTGGAAGCTCTGCTGGTTGA
Nfatc1	CCTGCTGCCTTACACAGTGCATTT	*Esm1*	GCAAGAGGACAGTGCTGGAT	*Col1a1*	GCTCCTCTTAGGGGCCACT
	TGCGACTATGAACAAGCCTACGGT		GGTGCCATAGGGACAGTCTTT		CCACGTCTCACCATTGGGG
Foxc1	GGAGATGTTCGAGTCACAGAGGA	*Angpt2*	AGAAAGTTCTGGACATGGAGGGCA	*Col3a1*	CTGTAACATGGAAACTGGGGAAA
	GACGTGCGGTACAGAGACTGG		TCTCCATTAGGTCATGCTGCTGCT		CCATAGCTGAACTGAAAACCACC
Foxc2	GAGTCCCAGGTGAGTGGCAAT	*Apln*	GACCGAGTTGCAGCATGAATC	*Col6a1*	CTGCTGCTACAAGCCTGCT
	ATTTCGTGCAGTCGTAGGAGTAGG		TCAAGGAGAGCCAGAGCAGC		CCCCATAAGGTTTCAGCCTCA
Mycn	AGCACCTCCGGAGAGGATAC	*Kcne3*	GACCAGGGCCAGACAATCAA	*Col6a2*	AAGGCCCCATTGGATTCCC
	ACGCACAGTGATCGTGAAAG		ACGTTTGTCCACTTTGCGTG		CTCCCTTCCGACCATCCGAT
Hey2	CGTCGGGATCGGATAAATAACA	*Mcam*	ATACCTGACTCCAGCCAAAC	*Postn*	GCTGCTGTTCCTGTGTGAT
	CAAGAGCGTGTGCGTCAAAG		ACAGCCACGATGACCACA		CCCGCAGATAGCACCTTGAT
Id2	CCAGAACAAGAAGGTGACCAAGATG	*Cav1*	ACACAGTTTCGACGGCATCT	*Olfml2a*	GAACTGCTTTCTGGTGTGCG
	AGGCTGACGATAGTGGGATGC		CATTGGGATGCCGAAGATCG		ACTGTGGGTGAGCATCAGTG
Tgfbi	ACCATGGACCGGATGTTGAC	*Trp53*	CATCACCTCACTGCATGGAC	*Twist1*	CAGCGGGTCATGGCTAAC
	GGCCACCAGCATGCTAAAAC		AAAAGATGACAGGGGCCATG		CAGCTTGCCATCTTGGAGTC
Ecm2	TGCTAGACTACCCACTGCCA	*Cdkn1a*	TTGTCGCTGTCTTGCACTCT	*Cdh2*	CTCCAGTCATAGGGAGCTGTC
	TCCATTTGAGCAGAGGCAGG		TTTCGGCCCTGAGATGTTCC		TCTTCTGAGACCTGGGTACAC
Ece1	CCCGCTGGAAGTTTTGTGTG	*Ccng1*	TGGCTTTGACACGGAGACATT	*Cdh1*	ATGGCCTTTCAAACACAGCCACAG
	TCTCCTTGGCTGATCTCCGA		GTCCAACACACCCAAGATGC		ACAATGACGTCCACCCTGTTCTCA
Jag2	CACGTGCATTGACGAAGTGG	*Gtse1*	AGAGGATCACCAGCAAGCTCCA	*Ltbp1*	ATGTGGGGAAGCCTTCTGTG
	ACCTTGCTACAATCCCGGTG		GTTTCGTCCTCTGAATGCTGGC		GCTGACGATCCACACCTGAA
Fabp4	CGATGAAATCACCGCAGACG	*Psrc1*	GAGTGTGCGCTGGAGGTCTAA	*Tab1*	ATCATCGCAGAGCCGGAAAT
	CCAGCTTGTCACCATCTCGT		GTCCAAGGTCTCGTCCACAAT		TACACGGTCTACCACAGCCT
Ddah1	TCCCTGTGGCCGATTCTTTG	*Vash1*	GAAGGTGAAGCTGGGTCAGT	*Map3k8*	ACATCGGCTCTGGTTTCGTT
	TCGGGTACAGTGAGCTTGTC		ATCTTGAGCCGCATGTCTCG		AGCAATGTTCTCATGCCGGA
Kitl	GCATGGAAGAAAACGCACCG	*Prdm16*	TGGGCTCACTACCCTACCAC	*Map3k6*	CGTGCACGGAGACTTTCACA
	CAATGCCATGGCTGTCCATT		GACTTTGGCTCAGCCTTGAC		TGTACCTTGTACATGCCCACC
Cd36	CTCATGCCAGTCGGAGACAT	*Myh7*	TGCAAAGGCTCCAGGTCTGAGGGC	*Adgrg6*	ACTGAGGCTGTGTGAAGTCG
	GCCACGTCATCTGGGTTTTG		GCCAACACCAACCTGTCCAAGTTC		ATTAGAGTGGAGCCGAGGGT
Plvap	GGCTAACCTGAGCAAACAGC	*Pdgfb*	AATGCTGAGCGACCACTCCA	*Blvrb*	GCACATGGAGTGGACAAGGT
	TCAGGATGATAGCGGCGATG		TACGGAGTCTCTGTGCAGCAGG		ATGTGGTCATCGGTCACGTC
Snail1	CGACTACCTAGGTCGCTCTG	*Pcdh17*	AAGCAAAACCTGGGCCCTTA	*Irx5*	TGGCTAAAGACCCGAAAATGT
	CTGCTGGAAGGTGAACTCCA		GAATGCACATCCGCGAAGAC		GGGATACCGCACCAGAGTTA
Snail2	CGAACTGGACACACACACAG	*Tgfb2*	AGGAGTGGCTTCACCACAAAGACA	*Tmem108*	GTACTCGCTGTCCACGAACC
	AAAGGAGAGTGGAGTGGAGC		ATTAGACGGCACGAAGGTACAGCA		AGAGTCTGGTAGGGCTGTGA
Zeb2	CACCCAGCTCGAGAGGCATA	*Dll4*	AGCTGGGTGTCTGAGTAGGC	*Flt4*	GGTTCCTGATGGGCAAAGG
	CACTCCGTGCACTTGAACTTG		AGAAGGTGCCACTTCGGTTA		TCAGTGGGCTCAGCCATAGG
Bmp10	GGAAGGGGACAACTGGAAAT	*Nppb*	ATCGGATCCGTCAGTCGTTT	*Odc1*	GGATGCGTTCTATGTTGCGG
	TGCTCCTTGTCATTGCTTTGG		CACTTCAAAGGTGGTCCCAGA		GTGCTCACTATGGCTCTGCT
Adm	CCTACGGGACTCGCTGATGA	*Hk2*	GGAACCCAGCTGTTTGACCA	*Slc2a1*	GGGCATGTGCTTCCAGTATGT
	TGGGCTTCGCTCTGATTGC		CAGGGGAACGAGAAGGTGAAA		ACGAGGAGCACCGTGAAGAT
Hif1	GCACGGGCCATATTCATGTC	*Vegfa*	TACCTCCACCATGCCAAGTG	*Epas1*	CACTGGCCCATGTCTTCCAT
	CACGTCATGGGTGGTTTCTTG		ACAGGACGGCTTGAAGATGTA		CAAGTGTGAACTGCTGGTGC
Igf2r	TCTGTGTTGGCTCGTCACTC	*Nrg1*	AGCTGGCCTGTAATTCTTCCTGT	*Eng*	TGCCCATTACCCTGCTGTTT
	CCGGTGACAGACGTTGATGA		CCAATGGCCACATTGCCAATAGGT		CGAAACGAGGACCAGGAACA
Tgfbr2	GAGTCGTTCAAGCAGACGGA	*Eno1*	CGCGTCTGTCCTTAAGGCTCTC	*Ptgs1*	CAGTATCACCTGCGGCTCTT
	GAACCAAATGGG GGCTCGTA		GCGGTGTACAGATCGACCTCA		CACACGCGGTTATGTTCACG
Tgfbr3	GGTGTGAACTGTCACCGATCA	*Wnt2*	ATGGCACTGGCTTCACTGTA	*Map2k6*	TTCTACGGTGCACTCTTCCG
	GTTTAGGATGTGAACCTCCCTTG		GGTGACGTGGGATGTGTCAT		GGCTTGACGTCTCGATGGAT
Ephb4	AGCGCTCTGGACAAGATGAT	*Smad9*	GTCTGACCTTGCAGATGGCT	*Map3k7*	ATAACACGCCGGAAACCCTT
	TGGTCCAAGAGTGGATGTGA		TAGGTGCCAGGCTGAGAGAT		GGTCCTTAGACCAACAGCGT
Aplnr	TTTGGGAAAGAGTGACTGGAGCCT	*Npr3*	GTCTACAGCGACGACAAACTC	*Tgfb3*	GATCACCACAACCCACACCT
	TGTCCAGAGCCCTCCAAGAACAAA		AGGTCCAAGTCTTTGGTCTCG		AGGTTCGTGGACCCATTTCC
β-actin	TTTGCAGCTCCTTCGTTGC				
	CATCCATGGCGAACTGGTG				

HUMAN	Primer	*HUMAN*	Primer	*HUMAN*	Primer
h HAND2	ATGAGTCTGGTAGGTGGTTTTCC	*h VEGFA*	GATGAGCTTCCTACAGCACAACAA	*h SOX17*	GTGGACCGCACGGAATTTG
	CATACTCGGGGCTGTAGGACA		TTTCGTTTTTGCCCCTTTCC		GGAGATTCACACCGGAGTCA
h CXCR4	ATCTGTGACCGCTTCTACCCC	*h EPHB2*	TATGCAGAACTGCGATTTCCAA	*h CD44*	CCTTGGCCACCACTCCTAAT
	AAACTGGAACACAACCACCCA		TGGGTATAGTACCAGTCCTTGTC		CCCTTCTGTCACATGGGAGTC
h JAG1	CAGATTTCCTTGTTCCCTTGCT	*h ESM1*	ACTTGCTACCGCACAGTCTCAG	*h ADM*	GAG TGG AAG TGC TCC CCA CTT
	CGTTGTTGGTGGTGTTGTCCT		AATCCATCCCGAAGGTGCCGTA		CTT GTA CCA TGG GCG CCT A
h PDGFB	GGCCGAGTTGGACCTGAACA	*h FLT4*	AGAGACTTTGAGCAGCCATTCATC	*h NRP1*	TTCAGGGCCATTTCTTTTTATC
	TCCAGCTCGCCTCCAGAGTG		GTCATCCCACACCACCTCCT		GGAACATTCAGGACCTCTCTTG
h SEMA3G	CTCAAAGTCATCGCTCTCCAGGC	*h ANGPT2*	AACTTTCGGAAGAGCATGGAC		
	AGTGCCGTAAGTCTCACATTGG		CGAGTCATCGTATTCGAGCGG		

Supplemental Table 5: Marker gene list

EC subtype	Marker genes
Arterial EC (12, 13)	*Gja5, Jag1, Cxcr4, Dll4, Unc5b, Sox17, Gja4, Hey1, Hey2, Ptp4a3, Kcnj8, Efnb2, Slc45a4*
Venous EC (12, 13)	*Aplnr, Nrp2*
Endocardial EC (14)	*Olfml3, Cdkn1c, H19, Adgrg6, Blvrb, Irx5, Igf2r, Tmem108, Bmx, Npr3, Nrk, Ece1, Plvap, Gsta4*
Tip cells (12, 13)	*Angpt2, Apln, Plxnd1, Adm, Mcam, Esm1, Dll4, Unc5b, Cxcr4, Igfbp3, Rasgrp3, Spry4, C1qtnf6, Kcnj8, Inhbb, Ptp4a3, Rhoc, Sox7, Guk1, Plaur, Nid2, Kcne3, Trp53i11, Spry1*
Endo-MT ECs (15-18)	*Zeb1, Zeb2, Cdh2, Serpine1, Col1a2, Snai2, Col5a1, Col6a3, Tgfbi, Tgfb2, Tgfb3, Tgfbr3, Olfml3, Ecm2, Postn, Tbx20, Acvr1, Thsd4, Tmem100, Ltbp1, Hand2, Twist1, Ecm1, Snai1, Nfatc1, Fbn1, Smtnl2, Gata6, Mfap4, Loxl, Col26a1, Col11a1, Col15a1, Pdgfrb, Smoc1, Id2.*

Supplemental references

1. Zhang YS, Xiong GF, and Peng JP. Effects of TGF beta-1 on mouse embryo implantation and expression of H2-D1 and H2-DM. *Front Biosci (Elite Ed).* 2010;2(1):351-60.
2. Zhao M, Zhang H, Liu X, Jiang Y, Ren L, and Hu X. The Effect of TGF-β on Treg Cells in Adverse Pregnancy Outcome upon Toxoplasma gondii Infection. *Front Microbiol.* 2017;8:901.
3. McGinnis CS, Murrow LM, and Gartner ZJ. DoubletFinder: Doublet Detection in Single-Cell RNA Sequencing Data Using Artificial Nearest Neighbors. *Cell Syst.* 2019;8(4):329-37 e4.
4. Yang S, Corbett SE, Koga Y, Wang Z, Johnson WE, Yajima M, et al. Decontamination of ambient RNA in single-cell RNA-seq with DecontX. *Genome Biol.* 2020;21(1):57.
5. Hao Y, Stuart T, Kowalski MH, Choudhary S, Hoffman P, Hartman A, et al. Dictionary learning for integrative, multimodal and scalable single-cell analysis. *Nat Biotechnol.* 2024;42(2):293-304.
6. Street K, Risso D, Fletcher RB, Das D, Ngai J, Yosef N, et al. Slingshot: cell lineage and pseudotime inference for single-cell transcriptomics. *BMC Genomics.* 2018;19(1):477.
7. Van den Berge K, Roux de Bezieux H, Street K, Saelens W, Cannoodt R, Saeys Y, et al. Trajectory-based differential expression analysis for single-cell sequencing data. *Nat Commun.* 2020;11(1):1201.
8. Finak G, McDavid A, Yajima M, Deng J, Gersuk V, Shalek AK, et al. MAST: a flexible statistical framework for assessing transcriptional changes and characterizing heterogeneity in single-cell RNA sequencing data. *Genome Biol.* 2015;16:278.
9. Andreatta M, and Carmona SJ. UCell and pyUCell: single-cell gene signature scoring for R and Python. *Bioinformatics.* 2026;42(2).
10. D'Amato G, Phansalkar R, Naftaly JA, Fan X, Amir ZA, Rios Coronado PE, et al. Endocardium-to-coronary artery differentiation during heart development and regeneration involves sequential roles of Bmp2 and Cxcl12/Cxcr4. *Dev Cell.* 2022;57(22):2517-32 e6.
11. Zeng Z, Ma Y, Hu L, Tan B, Liu P, Wang Y, et al. OmicVerse: a framework for bridging and deepening insights across bulk and single-cell sequencing. *Nat Commun.* 2024;15(1):5983.
12. Luo W, Garcia-Gonzalez I, Fernandez-Chacon M, Casquero-Garcia V, Sanchez-Munoz MS, Muhleder S, et al. Arterialization requires the timely suppression of cell growth. *Nature.* 2021;589(7842):437-41.
13. Cano E, Schwarzkopf J, Kanda M, Lindberg EL, Hollfinger I, Pogontke C, et al. Intramyocardial Sprouting Tip Cells Specify Coronary Arterialization. *Circ Res.* 2024;135(6):671-84.
14. Phansalkar R, Krieger J, Zhao M, Kolluru SS, Jones RC, Quake SR, et al. Coronary blood vessels from distinct origins converge to equivalent states

during mouse and human development. *Elife.* 2021;10.

15. Deshmukh AP, Vasaikar SV, Tomczak K, Tripathi S, den Hollander P, Arslan E, et al. Identification of EMT signaling cross-talk and gene regulatory networks by single-cell RNA sequencing. *Proc Natl Acad Sci U S A.* 2021;118(19).
16. de Soysa TY, Ranade SS, Okawa S, Ravichandran S, Huang Y, Salunga HT, et al. Single-cell analysis of cardiogenesis reveals basis for organ-level developmental defects. *Nature.* 2019;572(7767):120-4.
17. Lu P, Wu B, Wang Y, Russell M, Liu Y, Bernard DJ, et al. Prerequisite endocardial-mesenchymal transition for murine cardiac trabecular angiogenesis. *Dev Cell.* 2023;58(9):791-805 e4.
18. Yoshimatsu Y, and Watabe T. Emerging roles of inflammation-mediated endothelial-mesenchymal transition in health and disease. *Inflamm Regen.* 2022;42(1):9.
