# Supplementary material for: Endocardial transcription factor HAND2 orchestrates hypoxic and TGF-β signaling to modulate coronary artery formation
Source: J Clin Invest. 2026 Aug 6;136(19):e203310. doi: 10.1172/JCI203310 (PMC13626820; doi:10.1172/JCI203310)

# Original gel images of western blot

Fig.4o

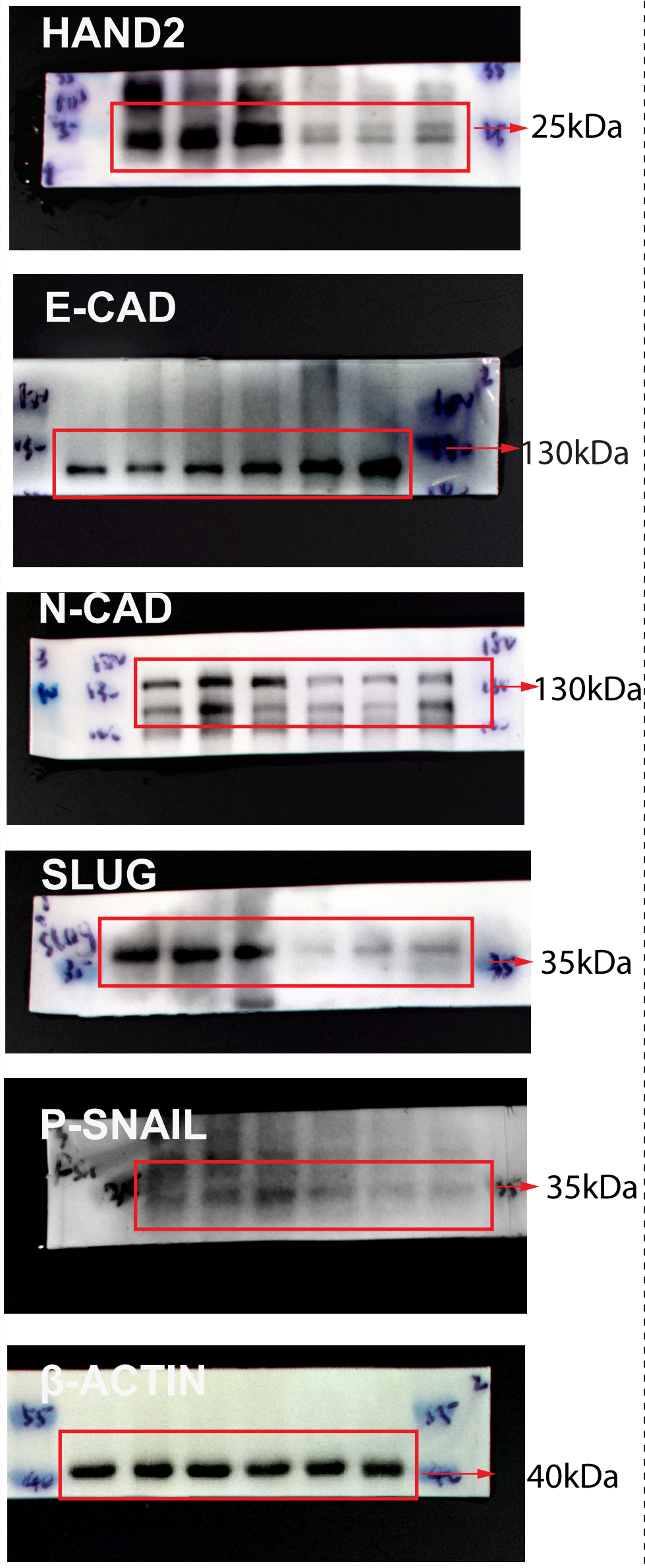


Fig.6g

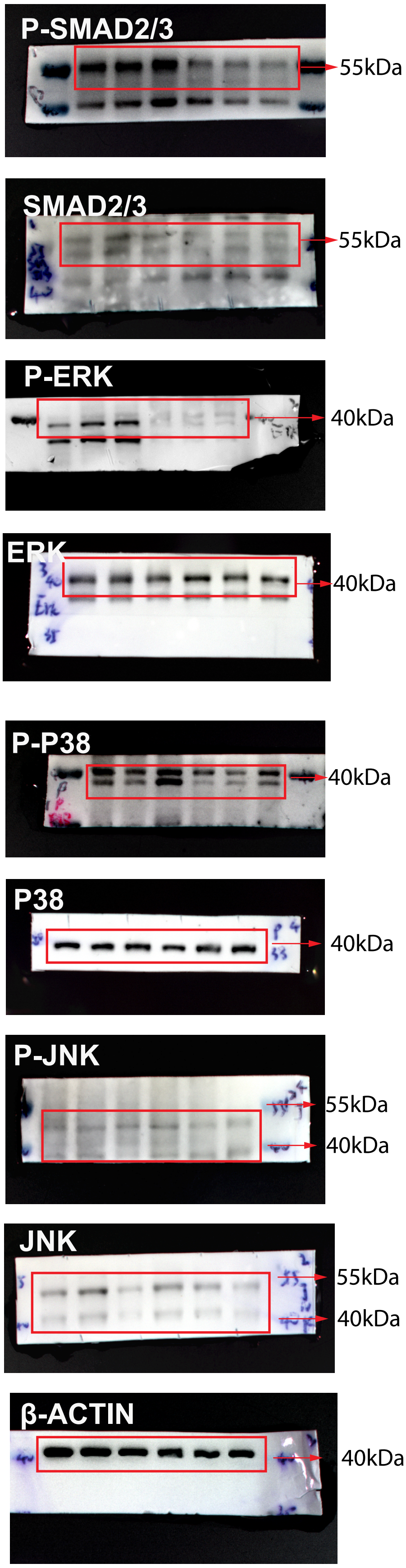


Supplementary Fig.7c

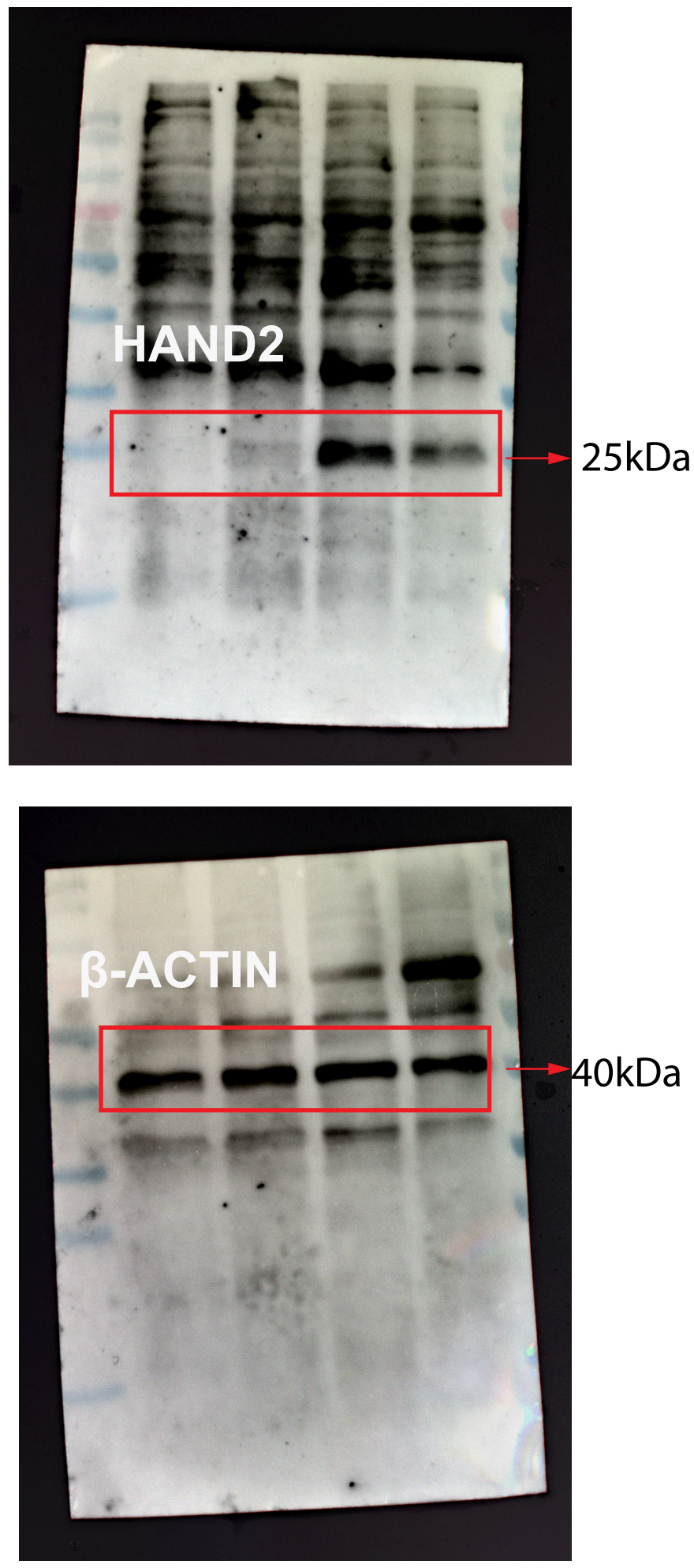

Supplement: Supplementary file 2 [file jci-136-203310-s142.pdf]
